# De novo detection of somatic mutations in high-throughput single-cell profiling data sets

**DOI:** 10.1038/s41587-023-01863-z

**Published:** 2023-07-06

**Authors:** Francesc Muyas, Carolin M. Sauer, Jose Espejo Valle-Inclán, Ruoyan Li, Raheleh Rahbari, Thomas J. Mitchell, Sahand Hormoz, Isidro Cortés-Ciriano

**Affiliations:** 1 European Molecular Biology Laboratory, European Bioinformatics Institute, Hinxton, Cambridge, UK; 2 Wellcome Trust Sanger Institute, Wellcome Genome Campus, Hinxton, Cambridge, UK; 3 Cambridge University Hospitals NHS Foundation Trust and NIHR Cambridge Biomedical Research Centre, Cambridge, UK; 4 Department of Surgery, University of Cambridge, Cambridge, UK; 5 Department of Systems Biology, Harvard Medical School, Boston, MA USA; 6 Department of Data Science, Dana-Farber Cancer Institute, Boston, MA USA; 7 Broad Institute of MIT and Harvard, Cambridge, MA USA

**Keywords:** Statistical methods, Cancer genomics, Genomic instability, Genetics research

## Abstract

Characterization of somatic mutations at single-cell resolution is essential to study cancer evolution, clonal mosaicism and cell plasticity. Here, we describe SComatic, an algorithm designed for the detection of somatic mutations in single-cell transcriptomic and ATAC-seq (assay for transposase-accessible chromatin sequence) data sets directly without requiring matched bulk or single-cell DNA sequencing data. SComatic distinguishes somatic mutations from polymorphisms, RNA-editing events and artefacts using filters and statistical tests parameterized on non-neoplastic samples. Using >2.6 million single cells from 688 single-cell RNA-seq (scRNA-seq) and single-cell ATAC-seq (scATAC-seq) data sets spanning cancer and non-neoplastic samples, we show that SComatic detects mutations in single cells accurately, even in differentiated cells from polyclonal tissues that are not amenable to mutation detection using existing methods. Validated against matched genome sequencing and scRNA-seq data, SComatic achieves F1 scores between 0.6 and 0.7 across diverse data sets, in comparison to 0.2–0.4 for the second-best performing method. In summary, SComatic permits de novo mutational signature analysis, and the study of clonal heterogeneity and mutational burdens at single-cell resolution.

## Abstract (web summary)

SComatic identifies somatic mutations from scRNA-seq and scATAC data without a matched reference sample.

## Main

Characterization of somatic mutations at single-cell resolution is essential to study genetic heterogeneity and cell plasticity in cancer^1^, clonal mosaicism in non-neoplastic tissues^2^ and to identify the mutational processes operative in both malignant and phenotypically normal cells^3,4^. Single-cell genome sequencing provides the most direct way to study mutations in single cells. However, single-cell genomics methods are not easily scalable and suffer from high rates of genomic drop-outs and artefacts introduced during whole-genome amplification^5^. To circumvent these issues, other approaches rely on bulk sequencing of single-cell-derived colonies grown in vitro or clonal populations directly isolated from tissues^6–8^. However, in vitro growth of single-cell-derived colonies is laborious and limited to cell types amenable to cell culture^5,7,9^, and isolation of clonal units is not technically feasible for some tissues. More recently, the development of ultra-sensitive sequencing methods using strand-specific barcoding has permitted the detection of mutations at single-molecule resolution^10,11^. Yet, cell type information is lost unless cell sorting is performed before sequencing. Due to these technical limitations, our understanding of the patterns of somatic mutations across cell types, and their impact on cell fates and phenotypes, remains limited.

An alternative strategy to single-cell genome sequencing consists of detecting somatic mutations in sequencing reads from high-throughput single-cell assays directly, such as scRNA-seq and scATAC-seq. The main advantage of this approach is the possibility to harness the high throughput of single-cell assays to map the lineage of cells to transcriptional or regulatory programs^12,13^ without the need for complex experimental protocols for joint profiling of the DNA and RNA from the same cell^3,8,14–16^. Nevertheless, the detection of mutations is strongly limited owing to the variability in gene expression across cell types, allelic drop-out events, RNA editing, limited depth of coverage and sequencing artefacts^17–19^. Therefore, existing algorithms rely on detecting mutations, such as single-nucleotide variants (SNVs) or indels, previously identified using matched bulk or single-cell DNA sequencing data^18,20–22^. These approaches are limited because matched DNA sequencing data are rarely available for existing high-throughput single-cell data sets, and sampling biases or genetic heterogeneity between the samples undergoing DNA sequencing and single-cell profiling can affect sensitivity for mutation detection. Therefore, algorithms designed to detect somatic mutations in single-cell data sets de novo without requiring matched DNA sequencing data are needed.

To address this need, we developed SComatic, an algorithm for de novo detection of somatic SNVs in single-cell profiling data sets without requiring matched bulk or single-cell DNA sequencing data. Using published scRNAs-seq data from 622 samples and scATAC-seq data from 66 samples, totaling 2,655,775 non-neoplastic and cancer cells (Supplementary Table 1), we show that SComatic has consistently higher precision compared to existing algorithms for somatic SNVs calling across diverse cancer data sets. In addition, we show that SComatic permits the detection of mutational burdens and de novo discovery of mutational signatures at cell-type resolution, even for differentiated cells and cells from polyclonal tissues showing high levels of genetic heterogeneity. SComatic is implemented in Python 3 and is available at https://github.com/cortes-ciriano-lab/SComatic.

## Results

### Overview of SComatic

**Fig. 1:**
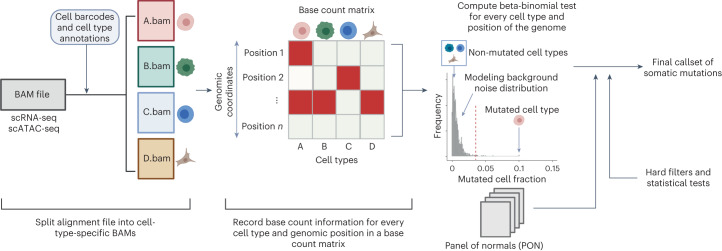
Overview of SComatic. Methodology for detecting somatic mutations in high-throughput single-cell profiling data sets. The dashed red line shows an arbitrarily chosen level of significance for illustration purposes.

We developed SComatic to detect somatic mutations using single-cell sequencing data without requiring a matched reference sample (Fig. 1). In brief, SComatic computes base counts for every position of the genome across cell types from the same individual using cell type annotations established through, for example, marker gene expression (Fig. 1 and Methods). Somatic mutations are distinguished from germline polymorphisms and artefacts using a set of hard filters and statistical tests (Fig. 1). Specifically, candidate somatic SNVs are distinguished from background sequencing errors and artefacts using a beta-binomial test parameterized using non-neoplastic samples (Methods). Next, mutations detected in multiple cell types are considered to be germline polymorphisms or artefacts and therefore discounted as somatic. The key idea is that germline variants should be present in all cell types, whereas somatic mutations should be detected only in cell types from the same differentiation hierarchy unless mutations were acquired in a progenitor or stem cell before clonal diversification or during early development^8,23,24^. Candidate mutations overlapping known RNA-editing sites or single-nucleotide polymorphisms (SNPs) with population frequencies greater than 1% in the gnomAD^25^ database are also filtered out. In addition, SComatic uses a panel of normals (PON) generated using a large collection of non-neoplastic samples to discount recurrent sequencing and mapping artefacts. For example, in 10× Genomics Chromium scRNA-seq data, recurrent errors are enriched in LINE and SINE elements, such as Alu elements (Supplementary Fig. 1), which are therefore not considered for mutation calling. Finally, to make a mutation call, SComatic requires a sequencing depth of at least five reads in the cell type in which the mutation is detected, and that the mutation is detected in at least three sequencing reads from at least two different cells of the same type (Supplementary Fig. 2 and Methods).

### Validation of SComatic using single-cell RNA-seq data

**Fig. 2:**
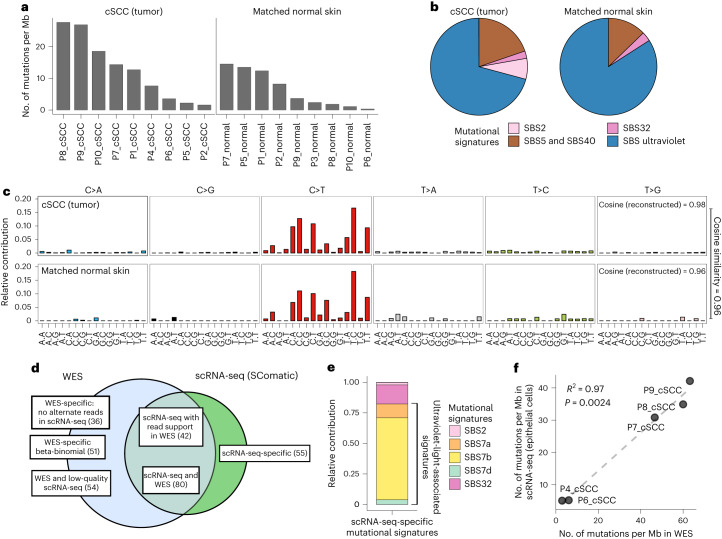
Validation of SComatic using matched scRNA-seq and exome sequencing data. **a**, Mutational burdens for epithelial cells using the somatic SNVs detected by SComatic in cSCC and matched normal skin scRNA-seq data sets. The number of mutations is normalized to account for the variable number of callable sites in each sample. **b**, Fraction of somatic SNVs detected in epithelial cells attributed to COSMIC signatures. SBS signatures associated with ultraviolet radiation (SBS7a, SBS7b, SBS7c and SBS7d) and clock-like mutational processes (SBS5 and SBS40) are collapsed for visualization purposes. **c**, Mutational spectra computed for the mutations detected using SComatic in epithelial cells from cSCC and matched normal skin scRNA-seq data. The cosine similarities between the observed and reconstructed mutational spectra are shown. **d**, Venn diagram showing the overlap of the somatic SNVs detected by SComatic in epithelial cells using scRNA-seq data and WES data from the cSCC samples. ‘WES-specific beta-binomial’ refers to mutations detected in WES with at least one alternative read count in scRNA-seq that are not significant for the beta-binomial test. **e**, Decomposition of the mutations detected in scRNA-seq data only (scRNA-seq-specific mutations) into COSMIC signatures. **f**, Correlation between the mutational burdens estimated using the mutations detected in WES and the mutations detected by SComatic in the scRNA-seq data. The correlation was assessed using a linear regression model. Only genomic regions with sufficient sequencing depth in both the WES and scRNA-seq data were considered for this analysis. Mb, megabase. Source data

To compare the patterns of mutations detected by SComatic against DNA sequencing data, we analyzed scRNA-seq data generated using the 10× Genomics Chromium technology and matched whole-exome sequencing (WES) data from eight cutaneous squamous cell carcinomas (cSCCs) and matched adjacent normal skin samples^26^. First, we compared the mutations detected by SComatic in epithelial cells with those detected by WES (Methods). For this analysis, we focused on the 9,788,377 positions in the genome across the eight samples with sufficient coverage in both the scRNA-seq and WES data (Fig. 2d and Methods). In these regions, we detected 266 of the 10,477 (2.4%) mutations found in the WES data, which we considered true positive mutations. Using SComatic, we detected 179 mutations in the scRNA-seq data (Fig. 2d), 80 (45%) of which were also detected in the WES data (Methods and Supplementary Tables 2 and 3). For 42 of the 179 mutations (23%), we found at least 1 read in the WES data supporting the mutated allele; however, this was insufficient evidence to call a mutation by our WES analysis pipeline (Methods). Finally, 55 of the 179 mutations (31%) were detected only in the scRNA-seq data. Of these 55 mutations, 38 (69%) were detected in sample P7. Of the 87 WES-specific mutations, 61 (70%) were also detected only in P7. Mutational signature analysis revealed that 45 (82%) of the mutations detected only in the scRNA-seq data and 71 (82%) of the WES-specific mutations were attributed to single-base substitution (SBS) mutational signatures linked with mutagenesis caused by exposure to ultraviolet radiation (SBS7a, SBS7b and SBS7d), which is consistent with the expected predominant signature for true mutations in these samples^26^ (Fig. 2e). The variant allele fractions of the mutations detected in WES and scRNA-seq data were not correlated for P7, unlike for other samples (Supplementary Fig. 3). Therefore, these results suggest that for sample P7, the lack of sequencing reads in the WES data supporting those mutations detected by SComatic in the scRNA-seq data (and vice versa) is probably due to high genetic heterogeneity.

Next, we applied SComatic to detect somatic mutations across all genomic positions with sufficient coverage in the scRNA-seq data (Methods). We detected 810 and 186 SNVs in the tumor and matched normal samples, respectively (Supplementary Table 1), which mapped to 3′ untranslated regions (40%), intronic (27%) and exonic regions (24%) (Supplementary Fig. 4). After normalizing by breadth of coverage (Methods), we estimated an average mutation rate per haploid genome for epithelial cells from the cSCC and normal skin samples of 12.8 and 3.7 mutations per Mb, respectively (note that we report mutational burdens for single cells as mutations per haploid genome because only one allele is generally detected per cell and genomic position; Supplementary Fig. 5). These rates are significantly higher than non-epithelial cells in the data set, which had a median of 0.33 and 0.40 mutations per Mb in tumor and matched normal samples, respectively (*P* < 0.001, Mann–Whitney *U*-test; Supplementary Fig. 6). Mutational signature analysis attributed 71% and 84% of the mutations detected in epithelial cells from tumor and matched normal skin samples, respectively, to signatures associated with exposure to ultraviolet radiation (SBS7a–d; Fig. 2b,c and Methods), consistent with previous DNA sequencing studies of somatic mutations in sun-exposed skin^7,27^. The remaining mutations were mostly attributed to SBS5 and SBS40 signatures (19.6% and 13.4% for the tumor and matched normal samples, respectively), which have been previously identified in non-neoplastic skin samples^7^. The mutation rates computed using the mutations detected in scRNA-seq data from epithelial cells were highly correlated with the rates estimated using the WES data (*R*^2^ = 0.97, *P* = 0.0024; Fig. 2f and Methods), indicating that SComatic permits the calculation of mutation burdens at cell-type resolution.

Together, these results show a high concordance between the mutations detected in scRNA-seq by SComatic and WES, and highlight that methods for calling mutations in single-cell data based on genotyping mutations previously identified in genome sequencing data are likely to have low sensitivity for samples showing high levels of genetic heterogeneity.

### SComatic outperforms existing mutation detection algorithms

**Fig. 3:**
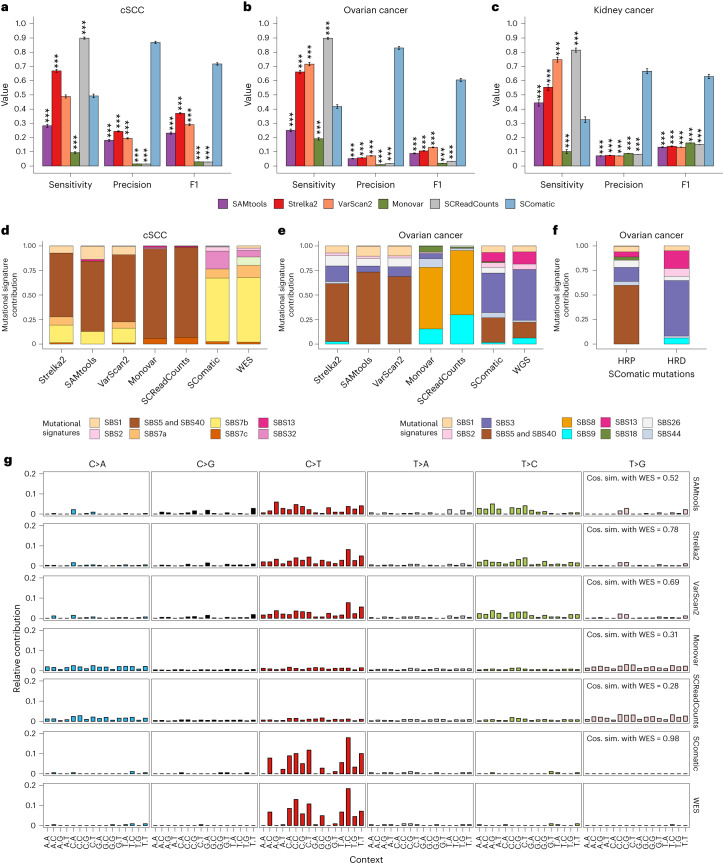
Comparison of the performance of SComatic against other mutation detection methods. **a**–**c**, Performance of Strelka2, SAMtools, VarScan2, Monovar, SCReadCounts and SComatic for the detection of somatic mutations in the scRNA-seq data from cSCC (**a**), ovarian cancer (**b**) and kidney tumor samples (**c**). The bars represent the mean value, and the error bars are the 95% bootstrap confidence interval for each statistic computed using 50 bootstrap resamples. Significance with respect to SComatic in **a**–**c** was assessed using the two-sided Student’s *t*-test (***P < 0.0001). **d**, Decomposition into COSMIC signatures of the mutations detected in cSCC scRNA-seq data and in matched WES data. **e**, Decomposition into COSMIC signatures of the mutations detected in scRNA-seq and matched WGS data from ovarian cancer samples. **f**, Decomposition into COSMIC signatures of the mutations detected by SComatic in scRNA-seq from homologous recombination deficient (HRD) and homologous recombination proficient (HRP) ovarian cancer samples. **g,** Comparison between the mutational spectra of the mutations detected in cSCC samples using WES and scRNA-seq data for the algorithms benchmarked. The cosine similarities between the mutational spectra computed using the mutations detected in the scRNA-seq and the WES data are shown. Source data

Next, we compared the performance of SComatic against top-performing pipelines developed for detecting somatic mutations in scRNA-seq data^22^ using popular variant calling algorithms (VarScan2 (ref. ^28^), SAMtools^29^ and Strelka2 (ref. ^30^)) and methods specifically designed for calling mutations in single-cell data (Monovar^31^ and SCReadCounts^32^). To this end, we used the matched genome sequencing and scRNA-seq data from epithelial cells from seven out of the eight cSCC tumors described above^26^, and from nine kidney and fourteen ovarian tumors^33,34^. In total, we considered 416 mutations detected in WES or whole-genome sequencing (WGS) data from 30 tumors with sufficient coverage in scRNA-seq data for benchmarking. We excluded patient P7 from the cSCC data set for this analysis owing to the high level of genetic heterogeneity observed between the matched scRNA-seq and WES data (Supplementary Fig. 3). To test whether the performance of SComatic is driven by filtering out common SNPs, we removed common polymorphisms from the mutation call sets generated by all algorithms used (Methods). SComatic achieved a sensitivity of 0.33–0.56 across the three data sets, which was higher than that achieved by SAMtools for two data sets and uniformly higher than that achieved by Monovar (Fig. 3a–c and Supplementary Table 4). Strelka2, VarScan2 and SCReadCounts showed a significantly higher sensitivity than SComatic (Fig. 3a–c). However, SComatic outperformed by a large margin all other methods in terms of precision across the three data sets: 0.67–0.87 for SComatic versus 0.06–0.24 for Strelka2, the algorithm with the next-best performance (*P* < 10^−15^, two-sided Student’s *t*-test; Fig. 3a–c and Supplementary Figs. 7 and 8). SComatic also achieved significantly higher F1 score values than other methods (Fig. 3a–c). Notably, we obtained similar differences in performance between methods when also including sample P7 from the cSCC data set in the benchmarking set (Supplementary Fig. 9).

To further compare the performance of these algorithms, we performed mutational signature analysis by fitting COSMIC signatures to the observed mutational spectra (Methods and Fig. 3d–g). We found that 77% of the mutations detected by SComatic in the cSCC data set were attributed to signatures SBS7a–d (*R* = 0.98 and *P* < 10^−15^; Fig. 3d and Supplementary Figs. 9 and 10), and the mutational spectrum was highly consistent with the WES data (cosine similarity = 0.98; Fig. 3g). By contrast, the mutations detected by the other algorithms were attributed to signatures SBS1 and SBS5 and were different from the patterns of mutations detected in WES (Fig. 3d). Similarly, the mutational signatures detected in the ovarian cancer data set using the mutations called in scRNA-seq by SComatic were highly concordant with the WGS data, in stark contrast to the results obtained with the other algorithms (Fig. 3e and Supplementary Figs. 9 and 10). Moreover, we found an enrichment of mutations attributable to mutational signature 3 (SBS3) in ovarian tumors with homologous repair deficiency (*n* = 22) compared to tumors with homologous repair proficiency (*n* = 13), which is consistent with previous studies of homologous repair deficient tumors^33,35,36^, highlighting the power of SComatic to detect clinically relevant mutational processes in scRNA-seq data (Fig. 3f and Supplementary Fig. 10).

Collectively, these results indicate that existing methods for detecting somatic mutations in scRNA-seq data have high false-positive rates, whereas SComatic enables the detection of somatic mutations at single-cell resolution at high precision. Moreover, these results illustrate that the higher performance of SComatic is not only driven by filtering out common SNPs but by the accurate modeling of the background error rate, which helps to distinguish artefacts from true mutations with high accuracy.

### Detection of somatic mutations in hypermutated samples

**Fig. 4:**
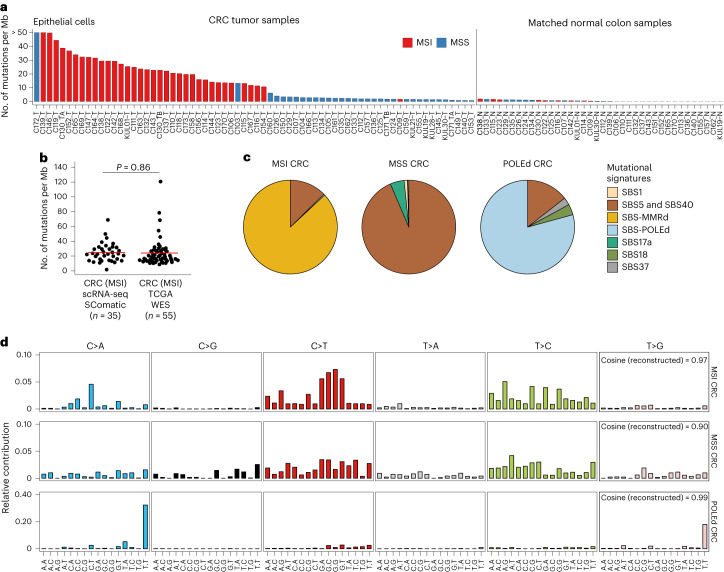
Detection of somatic mutations in scRNA-seq data from colorectal cancer samples. **a**, Mutational burden of epithelial cells computed using SComatic. The number of mutations is normalized to the number of callable sites per sample. **b**, Distribution of the mutational burden of epithelial cells from MSI tumors detected using SComatic and the mutational burden of MSI tumors from TCGA computed using WES data. The red horizontal line shows the mean for each group, and *n* indicates the number of samples per group. Statistical significance was assessed using the two-sided Student’s *t*-test. **c,** Decomposition of the mutational spectra computed using SComatic into COSMIC signatures. Mutational signatures associated with MMR deficiency (MMRd) (SBS6, SBS14, SBS15, SBS21, SBS26 and SBS44), POLE deficiency (POLEd) (SBS10a, SBS10b and SBS28) and clock-like mutational processes (SBS5 and SBS40) are collapsed for visualization purposes. **d**, Trinucleotide context of somatic mutations detected by SComatic using the scRNA-seq data from colorectal cancer samples. CRC, colorectal cancer; TCGA, The Cancer Genome Atlas. Source data

We next assessed the performance of SComatic to detect somatic mutations in samples characterized by a high mutational burden. To this end, we applied SComatic to scRNA-seq data from 70 treatment-naive primary colorectal tumors, including 37 mismatch repair (MMR)-deficient tumors showing microsatellite instability (MSI), and 40 matched normal adjacent colon samples^37,38^. Using SComatic, we called 8,997 somatic SNVs across all samples: 7,531 and 1,127 SNVs in MSI and microsatellite stable (MSS) tumors, respectively, and 339 in the matched normal samples (Supplementary Table 1). Most mutations mapped to non-coding elements, primarily untranslated regions (37%) and introns (27%) (Supplementary Fig. 4). Consistent with previous colorectal cancer genome studies^39,40^, our analysis revealed that epithelial cells in MSI tumors show a significantly higher mutational burden than epithelial cells from MSS tumors (24.7 vs 8.3 SNVs per Mb, *P* < 1.11 × 10^−12^; two-sided Mann–Whitney *U*-test) and normal adjacent colon samples (0.51 SNVs per Mb, *P* < 1.77 × 10^−15^). By contrast, the mutational burden for non-epithelial cells was low and comparable between MSI and MSS tumors (0.41 vs 0.52, *P* = 0.06; two-sided Mann–Whitney *U*-test), as expected for non-malignant cell types (Fig. 4a and Supplementary Fig. 6b). Moreover, the mutational burden estimated by SComatic using scRNA-seq data from epithelial cells in MSI tumors was comparable with that of MMR-deficient tumors estimated using WES data from The Cancer Genome Atlas^39,40^ (*P* > 0.05, Student’s *t*-test; Fig. 4b).

Mutational signature analysis attributed the mutations detected by SComatic in MSI tumors to SBS signatures associated with MMR deficiency (SBS6, SBS14, SBS15, SBS21, SBS26 and SBS44), SBS5 and SBS40 (Fig. 4c,d and Methods). In one sample (C172), 82.9% of mutations were attributed to signatures SBS10a, SBS10b and SBS28 (Fig. 4a,c,d), suggesting that hypermutation in this sample is driven by DNA polymerase epsilon (POLE) deficiency^41,42^. In MSS tumors, most mutations were attributed to signatures SBS5 and SBS40, consistent with published compendia of mutational signatures extracted from large cancer genome sequencing studies^42^.

We next compared the mutational burdens estimated by SComatic against VarScan2, SAMtools and Strelka2 using the colorectal cancer scRNA-seq data. As opposed to SComatic, the mutational burdens computed using the mutations detected by the other algorithms were not different between MSI or POLE-deficient and MSS or normal adjacent samples, consistent with the low specificity of existing methods for mutation calling using scRNA-seq data (Supplementary Fig. 11).

Together, these results indicate that SComatic permits the identification of the mutational processes operative in hypermutated samples at single-cell resolution without requiring matched genomic sequencing data.

### Detection of mutations in samples with a low mutation burden

**Fig. 5:**
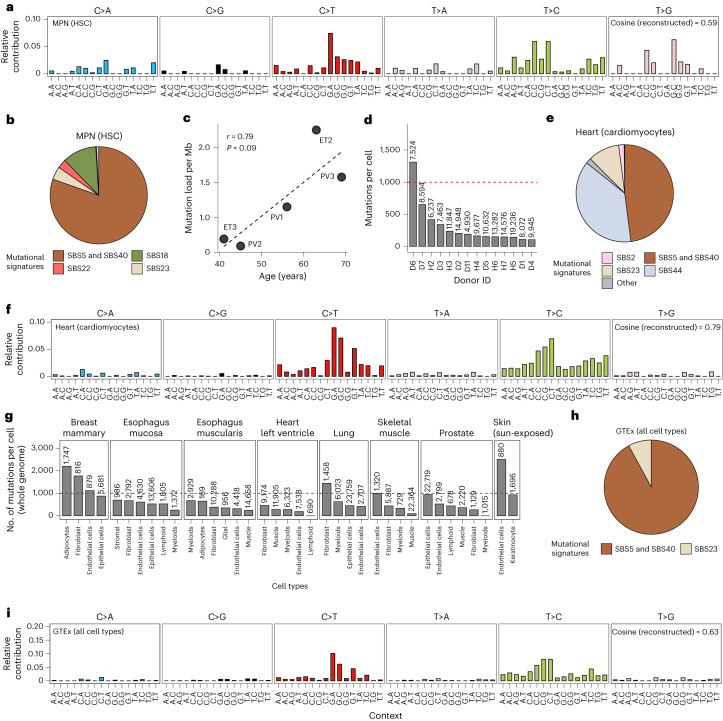
Detection of somatic mutations in samples with a low tumor mutational burden. **a**, Trinucleotide context of somatic mutations detected in HSCs from patients with MPNs. **b**, Decomposition of the somatic mutations detected in HSCs from patients with MPNs into COSMIC signatures. **c**, Correlation between the mutational burden of HSCs estimated using SComatic and the age of patients at the time of sampling (Pearson’s correlation test). **d**, Average number of mutations detected per cell and genome in cardiomyocytes from the heart cell atlas across donors. **e**, Decomposition of the mutations detected in cardiomyocytes into COSMIC signatures. **f**, Trinucleotide context of mutations detected in cardiomyocytes from the heart cell atlas. **g**, Average mutational burden of individual cells across the tissues included in the GTEx scRNA-seq data set. **h**, Decomposition of the mutations detected across all cells from the GTEx data set into COSMIC signatures. **i**, Trinucleotide context of mutations detected across all single cells from the GTEx data set. The numbers on top of the bars in **d** and **g** indicate the number of cells per cell type analyzed, and the horizontal red dashed line corresponds to 1,000 mutations per cell. Source data

We further tested the ability of SComatic to detect mutations in samples with low mutational burdens. To this end, we applied SComatic to scRNA-seq data from CD34^+^-enriched cells from five individuals with myeloproliferative neoplasms (MPNs), a type of blood cancer caused by the clonal expansion of a single hematopoietic stem cell (HSC)^8^. We detected an average of 0.12 mutations per Mb per haploid genome, which primarily mapped to intronic regions (62%; Supplementary Fig. 4). Mutational signature analysis revealed that 96% of the mutations detected by SComatic were attributed to signatures SBS5 and SBS40 (Fig. 5a,b), consistent with single-cell WGS studies of HSCs from healthy donors^6,43^ and MPN patients^8,44^. In addition, we found a positive correlation between the average mutation rate of HSCs estimated by SComatic and the patient’s age at the time of sampling (Pearson’s *r* = 0.79, *P* = 0.09; Fig. 5c), in agreement with previous studies^8^. Together, these results show that SComatic accurately detects mutational burdens and signatures in samples with low mutational burdens.

To further test whether SComatic can be used for the analysis of somatic mutations in non-neoplastic samples with high levels of genetic heterogeneity (for example, polyclonal tissues) and in differentiated cells, we next analyzed 10× scRNA-seq data from 78 samples obtained from six heart regions across 14 donors^45^. We detected a total of 2,132 somatic SNVs (Supplementary Table 1), 78% of which mapped to intronic regions (Supplementary Fig. 4). By extrapolating to the entire genome, we estimated an average mutation rate per haploid genome of 302 mutations for cardiomyocytes (range, 92–1,284; Fig. 5d), which was significantly lower than the mutation rates estimated for adipocytes (1,179 SNVs per cell and haploid genome) and smooth muscle cells (581; Supplementary Fig. 12a). Mutational signature analysis revealed that 46.7% of the mutations detected in cardiomyocytes were attributed to SBS5 and SBS40 (Fig. 5e,f), whereas 35.4% were attributed to SBS44, consistent with a recent study of somatic mutagenesis in human cardiomyocytes using single-cell genome sequencing^46^. The mutational burdens in cardiomyocytes estimated by SComatic were comparable to those estimated using single-cell WGS data^46^ (*P* = 0.08, two-sided Wilcoxon’s rank test; Supplementary Fig. 13).

Next, we applied SComatic to 24 scRNA-seq data sets from eight non-neoplastic tissues across 15 human donors generated by the GTEx consortium^47^. We found a total of 524 SNVs and estimated an average mutation load of 598 mutations per cell and haploid genome (Fig. 5g, Supplementary Fig. 12b and Methods). As observed in the heart cell atlas, adipocytes had the highest mutation burdens (1,430 mutations per cell and haploid genome), whereas muscle cells showed the lowest burdens (251; Supplementary Fig. 12b). As observed in other polyclonal tissues^7^, mutational signature analysis revealed that most of these mutations were attributed to the mutational signatures SBS5 and SBS40 (92.1%; Fig. 5h,i). Together, these results suggest that SComatic permits the study of the patterns and rates of mutations in polyclonal tissues.

### Performance of SComatic on single-cell ATAC-seq data sets

Next, we applied SComatic to detect somatic mutations using single-cell combinatorial indexing ATAC-seq (sciATAC-seq) data generated for 459,056 cells from 66 samples spanning 24 non-neoplastic tissues^48^. SComatic detected a total of 389 somatic SNVs (Supplementary Table 1). The distribution of mutations was different from those of scRNA-seq data sets, as most mutations mapped to intergenic (32%), promoter (19%) and intronic regions (18%) (Supplementary Fig. 4). We found low single-cell mutational burdens with an average load of 300 mutations per cell and haploid genome, with ductal cells showing the highest rates (933 per haploid genome), and skeletal myocytes (9 mutations) and follicular cells (0 mutations) having the lowest burdens (Supplementary Fig. 14a–c). As observed in other polyclonal tissues, 99% of the SNVs were attributed to SBS5 and SBS40 (Supplementary Fig. 14b,c). The genome-wide mutation rates were comparable to cell types represented in scRNA-seq and sciATAC-seq data sets, indicating that SComatic permits the estimation of mutation rates across different single-cell profiling assays (Supplementary Fig. 15).

### Patterns of clonality at cell-type resolution

Motivated by the importance of clonal mosaicism to somatic evolution and disease^2,49^, we next assessed whether the single-cell resolution provided by SComatic permits analysis of the patterns of clonality across cell types. To this end, we first computed the fraction of mutant cells per cell type across the single-cell data sets analyzed (Supplementary Table 1, Supplementary Fig. 13 and Methods). We detected clonal mutations in epithelial cells from the cSCC samples but not in epithelial cells from non-neoplastic skin samples, consistent with the high level of polyclonality in normal skin (Supplementary Fig. 16a,b). The clonality of mutations in epithelial cells in both MSI and MSS colorectal samples spanned a dynamic range of values, as expected for tumors harboring both clonal and subclonal mutations (Supplementary Fig. 16c,d). The mutations detected in non-neoplastic cell types from both cancer and non-neoplastic samples showed overall low (<0.2) mutant cell fractions, in agreement with genome sequencing studies of non-neoplastic tissues^7^ (Supplementary Fig. 16d–f). Together, these results show that SComatic permits the study of the clonality of mutations in both cancer and non-neoplastic samples.

### Analysis of intra-tumor heterogeneity using SComatic

**Fig. 6:**
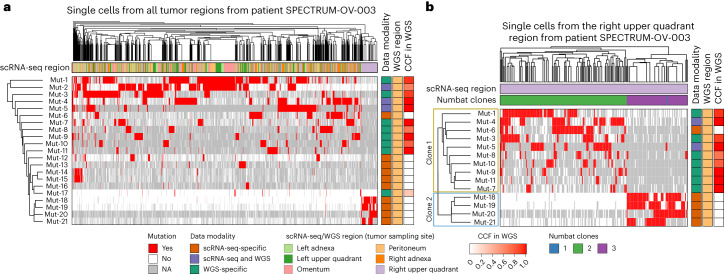
Analysis of intra-tumor heterogeneity using somatic mutations detected by SComatic in the scRNA-seq data from a patient with ovarian cancer (SPECTRUM-OV-003). **a**, Hierarchical clustering of single cells from all tumor regions (columns) by somatic mutations (rows; mutations are labeled arbitrarily). Mutations detected in the scRNA-seq data are shown in red. White denotes the absence of mutations in the scRNA-seq data in cases when the site was sufficiently covered (at least one sequencing read), and gray indicates that there was no coverage at the position to make a call. **b**, Hierarchical clustering of single cells collected from the upper right quadrant region from patient SPECTRUM-OV-003. Only the mutations shown in **a** that were detected in at least 20 cells are shown. The two clones defined by somatic mutations detected in scRNA-seq data are marked on the *y* axis. Single cells and mutations in **a** and **b** are ordered by hierarchical clustering (top and left-hand side dendrograms, respectively). The color bar indicates the cancer cell fraction (CCF) of the mutations in the WGS data. NA, no coverage in scRNA-seq. Source data

Next, we sought to evaluate whether mutations detected by SComatic in scRNA-seq data permit the reconstruction of the clonal heterogeneity in tumors. To this end, we analyzed multi-region scRNA-seq data from ovarian cancers (Methods). We used clones identified using copy number profiles inferred from scRNA-seq data using Numbat^50^ as a baseline for comparison (Methods). For example, for patient SPECTRUM-OV-003, we detected four mutations that were enriched in a subset of cells collected from the upper quadrant region (Fig. 6a and Supplementary Fig. 17). Unsupervised clustering using somatic mutations of the cells collected from this region revealed two clones. Clone 1 (marked in yellow in Fig. 6b) was defined by mutations detected by SComatic in a subset of cancer cells from the peritoneum and from other tumor regions. This is consistent with the fact that some of the SComatic mutations that define clone 1 were detected in the WGS data from the peritoneum. However, the mutations that define clone 2 (mutations 18–21; Fig. 6b) were detected only in scRNA-seq data in a subset of the cells from the upper right quadrant region. Clonal assignments based on somatic mutations and somatic copy number data were highly concordant (Fig. 6b and Supplementary Fig. 17). Together, these results show that SComatic permits, within the limits imposed by the breadth of coverage of current scRNA-seq methods, the reconstruction of the clonal heterogeneity of tumor samples and analysis of mutual exclusivity and co-occurrence of mutations at the single-cell level. Finally, SComatic also identified subclonal deleterious mutations in cancer driver genes frequently mutated in the cancer types analyzed, such as *MLH1, TGFBR2* and *KRAS* in colorectal tumors (Supplementary Fig. 18), indicating that SComatic can discover subclonal driver mutations.

### De novo mutational signature analysis

Clustering of samples based on the cosine similarity of their mutational spectra revealed groups consistent with the relative activity of known mutational processes in these samples (Supplementary Fig. 19). Therefore, we sought to determine whether the mutations detected by SComatic permit the identification of mutational processes using de novo mutational signature extraction. Decomposition of the mutations identified in epithelial cells from hypermutated colorectal cancer samples using COSMIC signatures revealed a strong contribution of signatures associated with POLE and MMR deficiency. By contrast, the signatures extracted from epithelial cells in MSS tumors showed strong contributions of SBS5 and SBS40, consistent with the mutational processes expected for these tumors (cosine similarities > 0.96; Supplementary Fig. 20). We identified two signatures in cSCC samples, one of which showed a cosine similarity of >0.98 when decomposed into the COSMIC signatures attributed to ultraviolet-light mutagenesis (SBS7a, SBS7b and SBS7c), and the other was decomposed into a combination of signatures SBS5 and SBS40, in agreement with the WES data (cosine similarity = 0.7; Supplementary Fig. 20). Despite the limited number of mutations and samples available for analysis (Supplementary Fig. 21), the signatures extracted from the mutations detected in non-neoplastic samples from the GTEx project and the heart cell atlas were decomposed into SBS5 and SBS40 (cosine similarity > 0.36; Supplementary Fig. 20), which is consistent with the mutational signatures identified in WGS studies of non-neoplastic samples^7^. The signatures detected in cardiomyocytes showed a strong contribution of SBS44, which is related to MMR deficiency and was reported in a recent single-cell WGS study of human cardiomyocytes^46^. Together, these results indicate that SComatic permits de novo mutational signature analysis using mutations detected in single-cell data.

## Discussion

Here, we show that SComatic permits de novo detection of somatic SNVs at single-cell resolution in single-cell data sets without requiring a matched reference sample. This is particularly relevant to the study of somatic mutagenesis in cell types and samples that cannot be reliably analyzed using existing single-cell genomics methods, such as differentiated cells and polyclonal tissues showing high levels of genetic heterogeneity^5,7^. Critically, we show that SComatic outperforms existing pipelines for the detection of somatic SNVs in single-cell data sets, which allows the identification of mutational processes in both cancer and non-neoplastic cells.

Despite its higher performance, SComatic is limited by the sparsity and low sequencing depth of current single-cell sequencing assays. As single-cell methods improve, SComatic will enable further insights to be derived from single-cell data sets, such as phylogenetic analysis and identification of mutations under positive selection driving clonal expansions in normal tissues and in cancer. Although somatic mutations can be detected in off-target regions, such as introns^51^, only a small fraction of the genome has sufficient sequencing coverage for detecting mutations reliably. Therefore, other methodologies are required to call mutations in regions missed by current scRNA-seq and ATAC-seq technologies or overlapping known RNA-editing sites.

The performance of SComatic is contingent on reliable cell type annotations, which can be challenging to obtain if clonally unrelated cells cannot be easily distinguished based on gene expression data alone^8,51^. In addition, the granularity of the cell type annotations used determines which types of mutations can be detected. Using very granular cell type annotations that consider, for example, two cell types from the same differentiation hierarchy as different cell types only permits the detection of somatic mutations acquired after clonal diversification, as mutations acquired in common progenitor or stem cells and during early development would be present in multiple cell types and therefore considered to be germline polymorphisms. By contrast, using broader cell type annotations encompassing, for example, multiple cell types from the same lineage permits the detection of mutations accumulated over longer periods of time, such as mutations acquired in the common lineage ancestors for the cell types grouped under a broad cell type category. Determining the granularity of the cell type annotations to be used depends on the biological question of interest. SComatic can easily be run using cell type annotations of variable granularity, thereby maximizing its applicability to the study of mutagenesis across development and disease evolution.

Overall, SComatic opens the possibility to study somatic mutagenesis in humans using single-cell data sets generated under the auspices of large-scale initiatives, such as the Human Cell Atlas or the Human Tumor Atlas Network^52,53^, as well as in other organisms.

## Methods

### Processing of single-cell data sets

The scRNA-seq data from cancer and non-neoplastic samples were downloaded in fastq format and processed uniformly. Specifically, raw sequencing reads were aligned to the GRCh38 build of the human reference genome using Cell Ranger^54^ v.6.0.1 and default parameter values to generate alignment files in binary alignment map (BAM) format and count matrices. Cell type annotations were downloaded from the original publications from which the data were downloaded (Supplementary Tables 1 and 5). Cell type annotations were used to assign sequencing reads to individual cells, and single cells without cell type annotations were discarded. Raw sciATAC-seq reads were mapped to the GRCh38 build of the human reference genome using BWA-MEM v.0.7.17-r1188 (ref. ^55^). In the case of sciATAC-seq data, aligned sequencing reads in BAM format were then processed following the Genome Analysis Toolkit (GATK) v.4.1.8.0 Best Practices workflow to remove duplicates and recalibrate base quality scores^56^.

### Detection of somatic mutations in single-cell data sets using SComatic

#### Processing of alignment files

In the first step of SComatic, the BAM file containing the sequencing reads for all cell types in a sample is split into cell-type-specific BAM files using precomputed cell type annotations (Supplementary Table 5). For this purpose, sequencing reads are assigned to individual cells using molecular barcodes (tag ‘CB’ in BAM files processed using Cell Ranger). Before identifying candidate mutation sites, reads with a mapping quality of lower than 255 (or 30 for sciATAC-seq data) or with more than five mismatches are filtered out. In addition, to ignore sequencing artefacts enriched in terminal ends of the reads or adapter sequences that were not properly trimmed, the base quality for the first five bases at the 3′ and 5′ ends of each read is set to 0 (ref. ^57^).

#### Collecting base count information

Next, the count of each base in each cell type for every position in the genome is recorded in a base count matrix indexed by cell types and genomic coordinates using the pileup functionality from the pysam version 0.21.0 module^58^. For this analysis, a minimum base quality of 30 is required, and only sites with a sequencing depth of five reads across at least two cell types are considered. Genomic positions overlapping RNA-editing sites are removed^59,60^. In addition, sites mapping to polymorphisms in the gnomAD^25^ database v.2.0.1 with a population frequency of greater than 1% are removed.

#### Detecting potential somatic SNVs

To distinguish technical artefacts, such as recurrent sequencing or mapping errors, from true somatic mutations, SComatic models the background error rate using a beta-binomial distribution. Specifically, non-reference allele counts at homozygous reference sites are modeled using a binomial distribution with parameter *P* (error rate), which is a random variable that follows a beta distribution with parameters α and β^57^. To infer the parameter values, SComatic uses base count information for one million sites in the genome, randomly selected from a panel of unrelated non-neoplastic samples generated using the same sequencing technology. Next, for each site in the genome and cell type, the beta-binomial distribution is used to test whether the non-reference allele counts are significantly higher than expected given the background error rate. Candidate somatic mutations are required to be present only in cells from the candidate cell type. To test this, SComatic requires that the beta-binomial test is not significant when applied to all other cell types independently and when applied to the base counts aggregated across all other cell types. The threshold for statistical significance for the beta-binomial is set to 0.001.

#### Filtering out recurrent artefacts

Owing to the enrichment of artefacts in repetitive regions (Supplementary Fig. 1) and the high error rate of Illumina sequencers at homopolymer tracts^61^, mutations mapping to or within 4 bp of mononucleotide tracts are removed. Finally, mutations mapping less than 5 bp apart from each other are filtered out. In this study, we applied this filter except for doublet base substitutions (DBSs) previously reported to be generated by specific mutational processes, such as CC > TT mutations associated with ultraviolet-light-induced mutagenesis in skin (COSMIC signature DBS1) and the characteristic DBS peaks observed in colorectal cancers (COSMIC signatures DBS2, 3, 4, 6, 7, 8, 10 and 11) (ref. ^42^).

In addition, SComatic generates a PON to discount positions affected by recurrent artefacts (sites with non-reference allele counts significantly higher than the background error rate modeled with the beta-binomial distribution). For this, SComatic uses a large collection of non-neoplastic data sets to assess the frequency of non-reference allele counts at each genomic site in the genome. This analysis serves to filter out candidate mutations mapping to regions of the genome prone to sequencing or mapping artefacts, germline variants missed by other filters and candidate mutations found in at least two unrelated samples, which are considered to be germline polymorphisms. The PONs generated in this study to call mutations in scRNA-seq and sciATAC-seq data are available at https://github.com/cortes-ciriano-lab/SComatic/tree/main/PoNs.

#### Calling somatic mutations

Finally, to make a mutation call, SComatic requires mutations to be supported by at least three reads from at least two cells from the same cell type. To tune this parameter, we performed mutational signature analysis on subsets of mutations defined based on the number of cells harboring each mutation. For this analysis, we focused on the somatic mutations detected by SComatic in epithelial cells from MSI tumors to guarantee sufficient statistical power for mutational signature analysis. We found that the mutational spectra and mutational signature contributions were consistent across subsets of mutations present in two or more cells (Supplementary Fig. 2), indicating that requiring mutations to be present in at least two cells to make a call is adequate to detect true somatic mutations.

### Estimation of mutational burdens

To compute the mutational burden at the cell-type level, we divided the total number of somatic mutations detected in each cell type by the total number of callable sites across all cells of the same type (Supplementary Fig. 21). Cell types with less than 500,000 callable sites were not included in this analysis. To estimate single-cell mutational burdens, we divided the number of mutations detected in each unique cell by the number of sites with a sequencing depth of at least one read and within the set of callable sites across all cells of the same type. We only considered the autosomes for computing mutational burdens. The sensitivity of single-cell assays to detect both alleles is low due to limited sequencing depth and allele-specific expression^17^. That is, we detect only one read per cell for most genomic positions in the genome. Therefore, our estimated mutational burdens for single cells mostly reflect the mutational burdens per haploid genome. For these reasons, we decided to report mutational burdens per haploid genome instead of correcting for ploidy because ploidy information for single cells was not available for the data sets analyzed. We note that not all cells analyzed might be diploid, as the data sets analyzed contained cell types that often undergo polyploidization, such as cancer cells and cardiomyocytes.

### Mutational signature analysis

Mutational signature analysis was performed using the R package MutationalPatterns^62^ and the COSMIC Mutational Signatures catalog v.3 (ref. ^42^). We used the function *fit_to_signatures* with default parameter values to estimate the contribution of selected mutational processes to the mutational spectrum observed in each sample. To account for differences in the frequency of each of the 96 trinucleotide contexts in which mutations can be detected between the whole genome and the regions profiled using scRNA-seq or sciATAC-seq, we normalized the frequency of mutations detected at each trinucleotide context. To do so, we first computed the frequency of each trinucleotide context in the human genome using the function *get_trinuc_norm* from the R package SigMA (https://github.com/parklab/SigMA). Next, for each single-cell data set, we estimated the frequency of each trinucleotide context across callable regions using a custom Python script, *TrinucleotideContextBackground.py*, which is provided as part of SComatic. To normalize the mutational spectra detected in each single-cell data set to the frequency of each trinucleotide in the whole genome, we divided the fraction of mutations detected at each trinucleotide context by the frequency of such context in the whole genome relative to its frequency in the single-cell data set being analyzed.

For fitting of COSMIC signatures, we used only the mutational processes known to be operative in each sample type analyzed^7,42^: (1) SBS1, SBS5, SBS6, SBS10a, SBS10b, SBS14, SBS15, SBS17a, SBS17b, SBS18, SBS21, SBS26, SBS28, SBS37, SBS40 and SBS44 for colorectal cancer samples; (2) SBS1, SBS2, SBS5, SBS7a, SBS7b, SBS7c, SBS7d, SBS13, SBS32 and SBS40 for cSCC samples; (3) SBS1, SBS2, SBS3, SBS5, SBS8, SBS9, SBS13, SBS18, SBS26, SBS40 and SBS44 for ovarian cancer samples^33^; and (4) SBS1, SBS2, SBS4, SBS5, SBS7a, SBS7b, SBS13, SBS16, SBS17b, SBS18, SBS22, SBS23, SBS32, SBS40, SBS41 and SBS88 for MPNs and non-neoplastic samples. We also included SBS6, SBS8, SBS19, SBS32, SBS35, SBS39 and SBS44 when analyzing heart samples^46^. The goodness of fit was determined by computing the cosine similarity between the observed and reconstructed mutational spectra using the estimated mutational signature contributions.

De novo mutational signature extraction was performed using non-negative matrix factorization as implemented in the R package MutationalPatterns using somatic SNVs detected in each of the following sample groups: epithelial cells from MSI and POLE-deficient colorectal cancer samples, epithelial cells from MSS colorectal cancer samples, epithelial cells from cSCC and matched normal skin samples, cardiomyocytes from the heart cell atlas and all cell types from the GTEx data set. The extracted signatures were decomposed into COSMIC v.3 signatures using the *fit_to_signatures* function after normalizing them to the trinucleotide frequencies of the whole genome. The goodness of fit of the decomposition of de novo signatures was estimated by computing the cosine similarity between the extracted mutational signature and the mutational spectrum reconstructed based on the estimated signature contributions.

### Whole-exome sequencing and whole-genome sequencing data analysis

Raw sequencing reads were mapped to the GRCh38 build of the human reference genome using BWA-MEM^29^ (v.0.7.17-r1188). Aligned sequencing reads in BAM format were processed to remove duplicates and recalibrate base quality scores following the GATK (v.4.1.8.0) Best Practices workflow^63^. Somatic SNVs in WES data from cSCC samples were detected using Strelka2 (ref. ^30^) (v.2.9.10) and MuSE^64^ (v.1.0rc) using default parameter values and the matched normal samples as germline controls. For benchmarking purposes, we considered only those somatic mutations detected by both algorithms. In the case of WGS data, somatic SNVs were detected using SAGE (v.2.8), and purity, ploidy and somatic copy number aberrations were estimated using PURPLE (v.2.54). Both SAGE and PURPLE are available at https://github.com/hartwigmedical/hmftools. Cancer cell fractions were computed as previously described^65^. Somatic SNVs detected in the kidney tumors used for benchmarking were downloaded from ref. ^34^.

### Comparison of mutations detected in scRNA-seq and genome sequencing data

To compare the mutations detected using matched genome sequencing (WES–WGS) and scRNA-seq data, we computed the base counts for all positions in the genome using the WES–WGS data. For this analysis, we focused only on regions with a coverage of at least 50× in the WES–WGS data from the cancer sample and 10× in the matched normal sample. In the case of the scRNA-seq data, we considered only regions with a sequencing depth of at least ten reads in cell types labeled as malignant, and with a depth of five reads in at least two additional cell types. Only regions that passed these filtering criteria for both the scRNA-seq and WES–WGS data were considered for benchmarking purposes. For the kidney and ovarian cancer data sets, only tumor regions with matched WES–WGS and scRNA-seq data were included in the benchmarking analysis. In the case of the ovarian cancer data set, WGS and scRNA-seq data sets were matched by *spectrum_sample_id*, and only scRNA-seq data sets with at least 100 ovarian cancer cells were considered. For benchmarking purposes, we considered only the 416 somatic mutations called in WES–WGS by mutation detection algorithms and with evidence of the mutation in the scRNAs-seq data: at least one read supporting the mutation in scRNA-seq reads from malignant cells with a base quality of ≥30 (Supplementary Table 3).

As we treated the WES–WGS data as the baseline for comparison, we categorized the mutations as: (1) true positives (mutations called in the scRNA-seq data by the algorithms benchmarked and in WES–WGS); (2) true positives with low support in WES–WGS (mutations called in the scRNA-seq data but not called by the WES–WGS mutation detection pipeline that nevertheless have at least one read supporting the mutant allele in WES–WGS with a base quality ≥ 30 and no reads supporting any other alternative allele); (3) false negatives (mutations called in WES–WGS that are not called in scRNA-seq); and (4) false positives (mutations called in the scRNA-seq data with no reads supporting the mutant allele in the WES–WGS data, and mutations called in scRNA-seq that nevertheless have read support for the mutant allele in the matched normal WES–WGS data, thus suggesting germline contamination).

To compute performance metrics, we estimated the sensitivity, precision and F1 score values for each algorithm using 50 bootstrap resamples generated by sampling with replacement from the set of mutations used for benchmarking. We then compared the performance between callers using the Student’s *t*-test, correcting for multiple hypothesis testing using the false discovery rate method. The calculation of the precision, sensitivity and F1 score values was performed as follows:

$${\rm{Precision}}=\,\frac{{{\rm{TP}}}_{{\rm{prec}}}}{{{\rm{TP}}}_{{\rm{prec}}}+{\rm{FP}}}$$

where TP_prec_ corresponds to the number of true positives and true positives with low support in WES–WGS, and FP is the number of false positives;

$${\rm{Sensitivity}}=\,\frac{{{\rm{TP}}}_{{\rm{sens}}}}{{{\rm{TP}}}_{{\rm{sens}}}+{\rm{FN}}}$$

where TP_sens_ corresponds to the number of true positives and FN is the number of false negatives; and

$${\rm{F}}1=\,\frac{{2\times {\rm{TP}}}_{{\rm{prec}}}}{{2\times {\rm{TP}}}_{{\rm{prec}}}+{\rm{FP}}+{\rm{FN}}}$$

### Detection of somatic mutations using existing algorithms

We compared the performance of SComatic against five different algorithms: Strelka2 (ref. ^30^), SAMtools (ref. ^66^), VarScan2 (ref. ^67^), Monovar^31^ and SCReadCounts^32^. Strelka2, SAMtools and VarScan2 were run using default parameter values^22^, and SCReadReadCounts was run using the discovery mode (*varLoci*) and requiring at least 3 supporting reads to make a call (*min_var_read_count* = 3), which is the minimum number of reads required by SComatic to make a call. All other parameters were set to default values. Strelka2, SAMtools, VarScan2 and SCReadReadCounts were run on cell-type-specific BAM files containing only the sequencing reads from the malignant cells: epithelial cells (cSCC data set), ovarian cancer cells (ovarian cancer data set) and renal cancer cells (kidney cancer data set). Cell-type-specific BAM files were generated using the Python script *SplitBamCellTypes.py*, which is available at https://github.com/cortes-ciriano-lab/SComatic/blob/main/scripts/SplitBam/SplitBamCellTypes.py. Monovar was run in multi-cell-type calling mode, which allows joint processing of multiple cell types from the same individual, and using default parameter values except for the mapping quality filter, which was set to 255 because this threshold corresponds to the standard mapping quality filter for scRNA-seq data. To detect mutations in only the malignant cell types of interest, we set the parameter -c to zero. Finally, the resulting mutation call sets generated using the five algorithms compared against SComatic were processed to filter out common SNPs reported in gnomAD (v.2.0.1)^25^ or ExAC (v.0.3)^68^ with a population frequency >1%, or present in a PON generated using WGS data from the 1000 Genomes Project (https://gatk.broadinstitute.org/hc/en-us/articles/360035890631-Panel-of-Normals-PON-).

### Analysis of the clonal architecture of tumors using somatic SNVs

To reconstruct the clonal structure of ovarian cancers using mutations detected in scRNA-seq data, we first ran SComatic on the scRNA-seq data from each tumor region. We did not run SComatic using all scRNA-seq data given that region-specific mutations present in a subset of cancer cells would not pass the threshold used in the beta-binomial test to distinguish true mutations from background errors. Next, all single cells from all regions were genotyped for the somatic mutations detected with SComatic in any of the regions analyzed. Specifically, cells were considered to be mutated if at least one read supporting a mutation detected by SComatic in the same or other region was present. This step was performed using a custom Python script (https://github.com/cortes-ciriano-lab/SComatic/blob/main/scripts/SingleCellGenotype/SingleCellGenotype.py). Finally, we recorded the presence or absence of each mutation in all single cells in a binary matrix, on which we performed unsupervised hierarchical clustering. For this analysis, we only considered cases in which we could detect mutations present in at least 20 cells and that could be genotyped in at least 10% of the cells across all tumor regions.

### Analysis of the clonal structure of tumors using Numbat

We used Numbat version 1.2.2 (ref. ^50^) to detect cancer cell clones based on copy number profiles inferred from the scRNA-seq data. SNP pileup data were generated using cellsnp-lite version 1.2.3 (ref. ^69^) and phased using Eagle2 (ref. ^70^) and the high polymorphic regions detected using WGS data from the 1000 Genomes Project mapped to the GRCh38 build of the human reference genome, as described in the Numbat documentation (https://github.com/kharchenkolab/numbat). Next, we ran Numbat using default parameter values and the processed allele count data together with the scRNA-seq expression data as the input. Samples for which sufficient somatic copy number events were detected for phylogeny construction and clonality inference were used for downstream analysis.

### Discovering clinically relevant driver mutations

Driver mutations were defined as those SNVs detected in tier-1 driver genes from The Cancer Gene Census catalog (version from July 2022)^71^ and predicted to be deleterious by MetaLR or MetaSVM, as implemented in Annovar (v.2018Apr16)^72^.

### Reporting summary

Further information on research design is available in the Nature Portfolio Reporting Summary linked to this article.

## Online content

Any methods, additional references, Nature Portfolio reporting summaries, source data, extended data, supplementary information, acknowledgements, peer review information; details of author contributions and competing interests; and statements of data and code availability are available at 10.1038/s41587-023-01863-z.

### Supplementary information

Supplementary Information Supplementary Figs. 1–21

Reporting Summary

Supplementary Table Supplementary Tables 1–5

### Source data

Source Data Fig. 2 Raw data for the plots visualized in Fig. 2a–f, including the bar plots, mutational spectra, mutational signatures and Venn diagram

Source Data Fig. 3 Raw data for the plots visualized in Fig. 3a–g, including the bar plots showing the results of the benchmarking analysis, and the mutational signature analyses

Source Data Fig. 4 Raw data for the plots visualized in Fig. 4a–d, including the bar plots, distributions, pie charts and mutational spectra

Source Data Fig. 5 Raw data for the plots visualized in Fig. 5a–i, including the bar plots showing the results of the benchmarking analysis, and the mutational signature analyses

Source Data Fig. 6 Data displayed in the heatmaps

## Data Availability

The raw WES and scRNA-seq data from the cSCC and matched normal samples are available in the Gene Expression Omnibus (GEO) database under accession number GSE144240. The raw scRNA-seq data from patients with MPN and colorectal cancer are available through controlled access application via dbGaP under dbGaP study accession numbers phs002308.v1.p1 and phs002407.v1.p1, respectively. The cell type annotations for the colorectal cancer data set^37^ are available in the GEO database under accession number GSE178341. Raw sequencing data and cell type annotations for six additional patients with colorectal cancer^38^ included in this study are available in the GEO database under accession number GSE144735. The cell type annotations for the MPN data set were obtained from our previous study^8^. Raw WES and scRNA-seq data from the kidney cancer samples are available at the European Genome-Phenome Archive (EGA) under accession numbers EGAD00001008029 and EGAD00001008030, respectively. Cell type annotations can be accessed at https://data.mendeley.com/datasets/g67bkbnhhg/1. Instructions to access the WGS data, scRNA-seq data and somatic copy number calls from the ovarian cancer data set are available at https://www.synapse.org/#!Synapse:syn25569736/wiki/612269. The raw scRNA-seq data and cell type annotations for the human heart cell atlas^45^ were downloaded from the Human Cell Atlas Data Coordination Platform with accession number ERP123138 (https://www.ebi.ac.uk/ena/browser/view/ERP123138). Cell type annotations were downloaded from the Human Cell Atlas Data Portal (https://data.humancellatlas.org/explore/projects/ad98d3cd-26fb-4ee3-99c9-8a2ab085e737). The raw scATAC-seq data and cell type annotations used in this study are available in the GEO database under accession number GSE184462. The raw sequencing data from GTEx samples are available at the Analysis Visualization and Informatics Lab-space (AnVIL; https://anvil.terra.bio/#workspaces/anvil-datastorage/AnVIL_GTEx_V9_hg38) and can be downloaded through controlled data access application via dbGaP under study accession number phs000424. The gnomAD database (v.2.0.1) and the ExAC data set (v.0.3) were downloaded from https://gnomad.broadinstitute.org. The COSMIC mutational signatures (v.3) were downloaded from https://cancer.sanger.ac.uk/signatures. The Cancer Gene Census (GCG) catalog (version from July 2022) was downloaded from https://cancer.sanger.ac.uk/census. The GATK PON generated using WGS data from the 1000 Genomes Project was downloaded from https://gatk.broadinstitute.org/hc/en-us/articles/360035890631-Panel-of-Normals-PON-. The DARNED database was downloaded from https://darned.ucc.ie. The REDIportal Database was downloaded from http://srv00.recas.ba.infn.it/atlas/download.html. Exome sequencing data from The Cancer Genome Atlas are available through controlled data access via dbGaP with accession number phs000178.v11.p8 and were downloaded from the Genomic Data Commons (GDC) Data Portal (https://portal.gdc.cancer.gov). The reference genome used in this study (GRCh38) was downloaded from https://hgdownload.soe.ucsc.edu/downloads.html. Source data are provided with this paper.

## Code availability

SComatic is available at https://github.com/cortes-ciriano-lab/SComatic.
