# Supplementary material for: De novo detection of somatic mutations in high-throughput single-cell profiling data sets
Source: Nat Biotechnol. 2023 Jul 6;42(5):758–67. doi: 10.1038/s41587-023-01863-z (PMC11098751; doi:10.1038/s41587-023-01863-z)
Supplement: Supplementary file 1 — Supplementary Figs. 1–21 [file 41587_2023_1863_MOESM1_ESM.pdf]

nature biotechnology

Article

https://doi.org/10.1038/s41587-023-01863-z

De novo detection of somatic mutations in high-throughput single-cell profiling data sets

In the format provided by the authors and unedited

Supplementary Figures

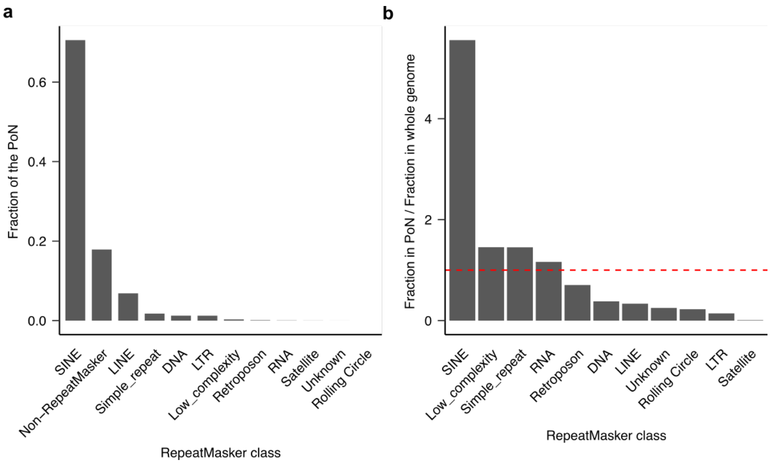

Supplementary Figure 1. Recurrent artefacts are enriched in repetitive elements. (**a**) Genomic distribution of artefactual sites included in the "Panel of Normals" (PoN) generated using scRNA-seq data across different types of repetitive elements. (**b**) Enrichment of artefactual sites in repetitive element classes. The dashed red line indicates no enrichment or depletion.

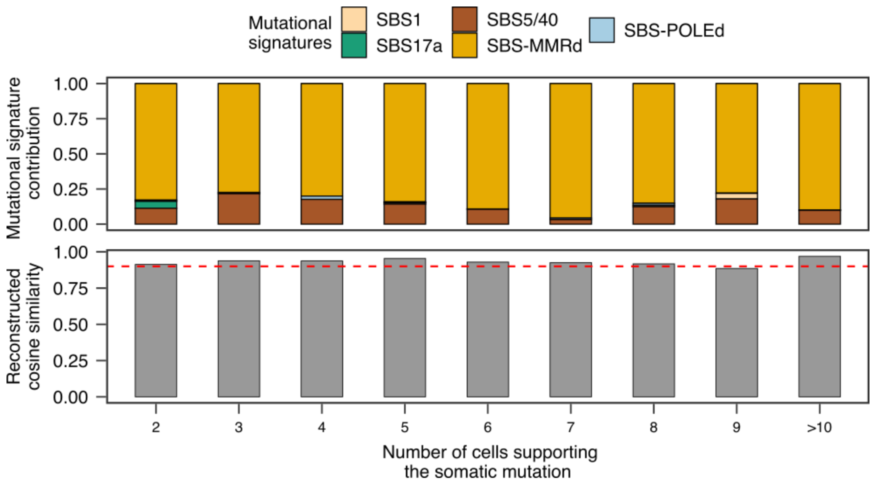

Supplementary Figure 2. Mutational signature analysis of the somatic mutations detected across an increasingly larger number of cells. Decomposition into COSMIC signatures of the somatic mutations detected in the MSI colorectal cancer data sets across increasingly higher cut-off values for the number of cells required to harbour a mutation to make a call. Overall, the contribution of mutational signatures associated with MMRd is constant across increasingly stringent cut-off values, indicating that requiring mutations to be detected in at least 2 cells to make a call is adequate to discover true somatic mutations. Mutational signatures associated with MMRd (SBS6, SBS14, SBS15, SBS21, SBS26 and SBS44), POLE-deficiency (SBS10a, SBS10b and SBS28) and clock-like mutational processes (SBS5 and SBS40) are collapsed for visualization purposes.

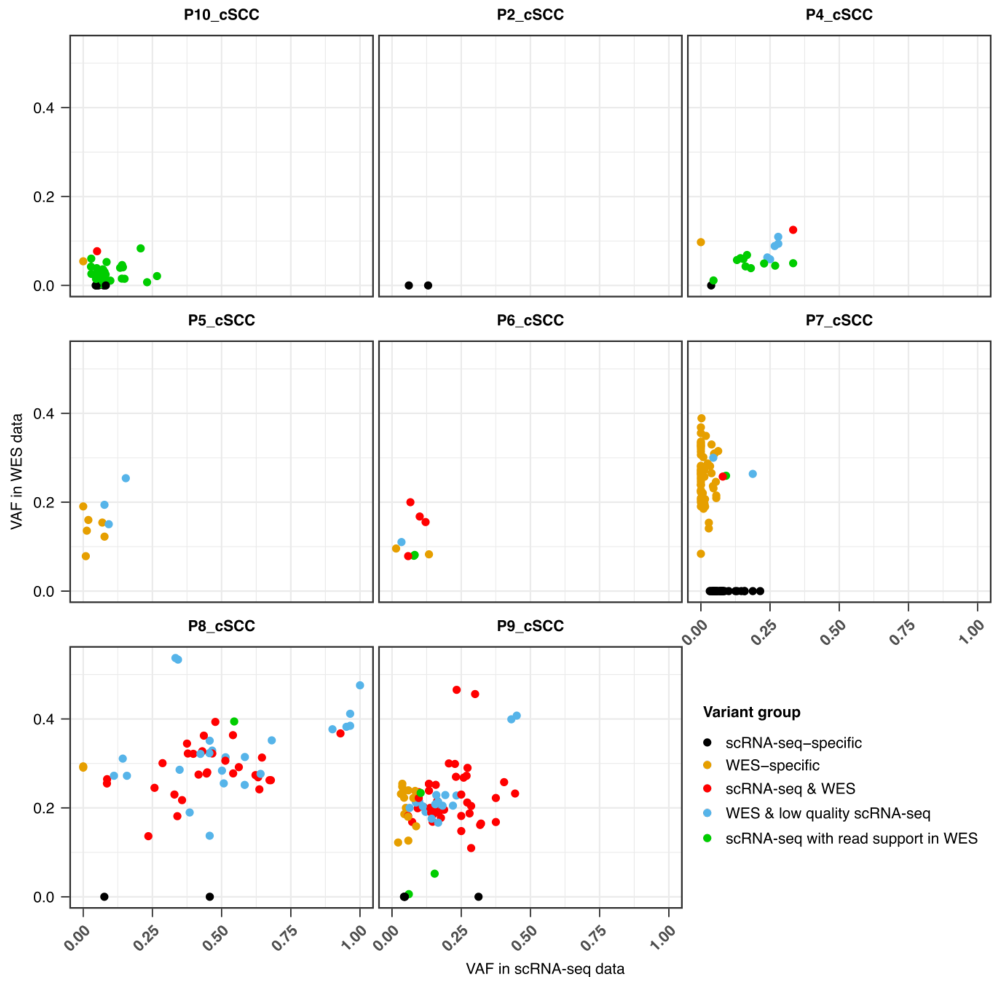

Supplementary Figure 3. Comparison of the variant allele fraction (VAF) of mutations detected in WES and scRNA-seq data from epithelial cells using SComatic.

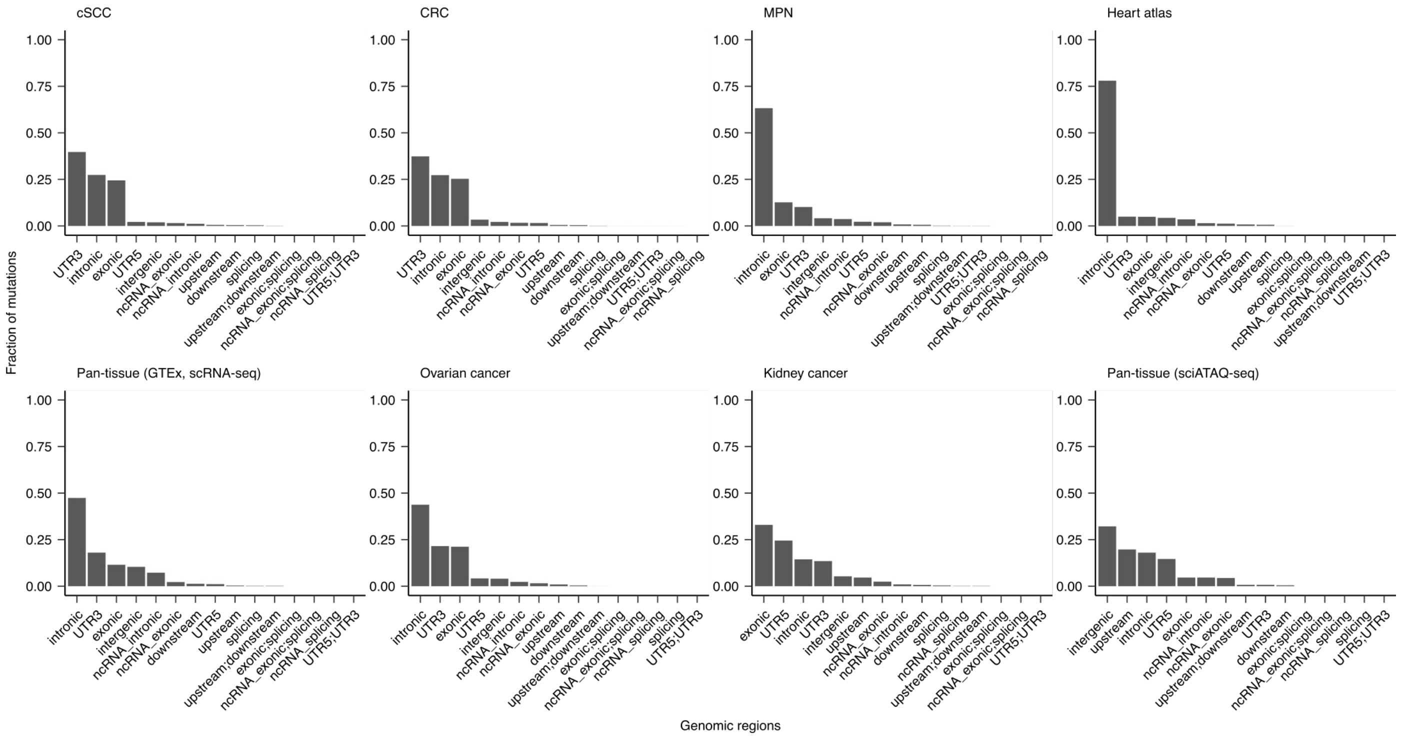

Supplementary Figure 4. Genomic distribution of somatic mutations detected by SComatic in single-cell data sets.

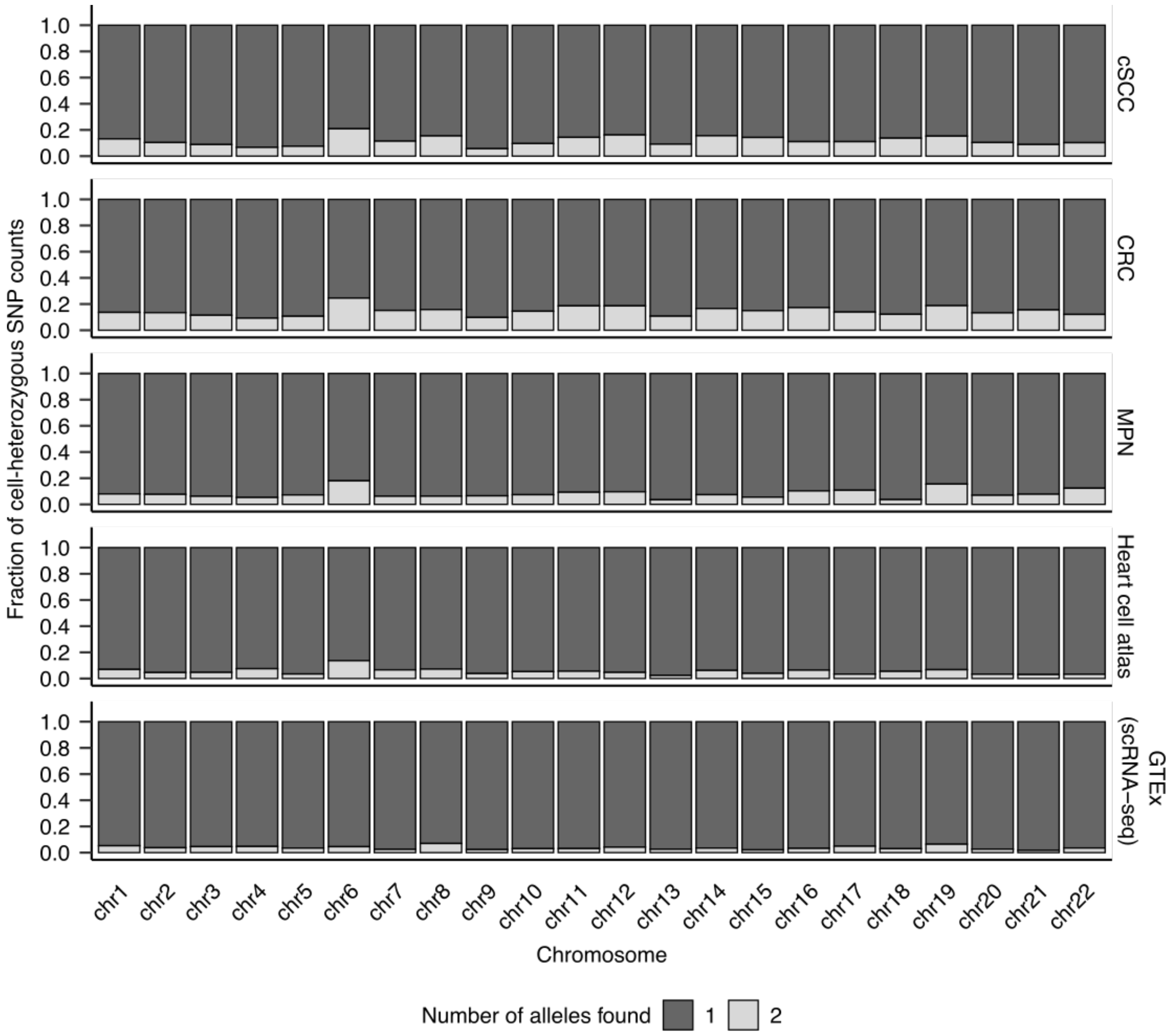

Supplementary Figure 5. Fraction of cell-heterozygous SNP pairs showing monoallelic (dark grey) or biallelic (light grey) expression. Heterozygous SNPs were identified as those common SNPs (MAF>10% in gnomAD) for which the alternate allele was detected in 25-75% of the scRNA-seq reads from each data set, requiring that each SNP was covered by >= 5 reads in at least two cell types.

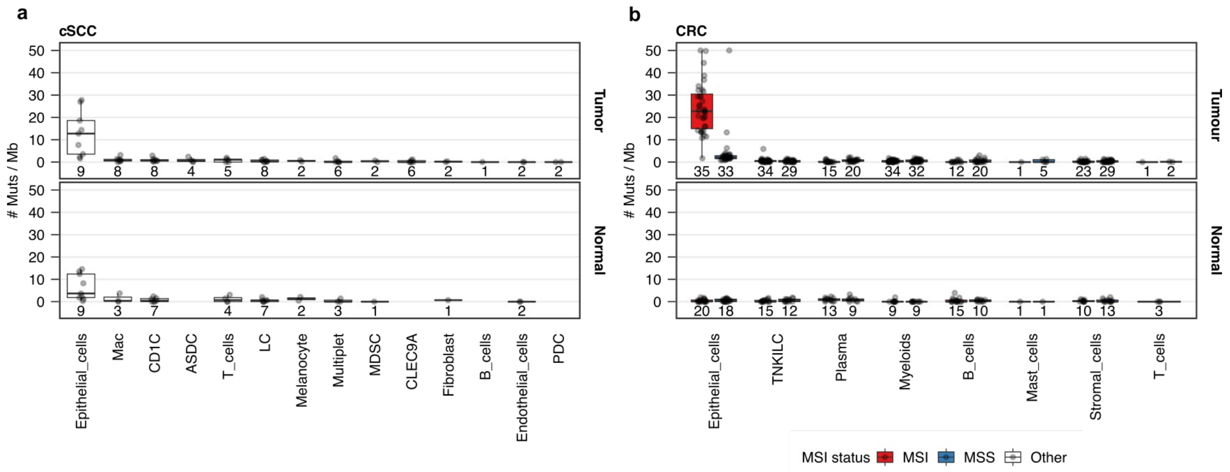

Supplementary Figure 6. Mutational burden across cell types. (**a**) Mutational burden across cell types detected in the scRNA-seq data from cSCC and matched normal skin samples. (**b**) Mutational burden across cell types detected in the colorectal cancer (CRC) and matched normal colon samples. Mac: macrophages; ASDC: AXL+SIGLEC6+ dendritic cells; LC: Langerhans cells; MDSC: myeloid-derived suppressor cells; PDC: Plasmacytoid dendritic cells; TNKILC: T-cells, natural killer cells, Innate lymphoid cells. Box plots show median, first and third quartiles (boxes), and the whiskers encompass observations within a distance of 1.5× the interquartile range from the first and third quartiles. The number of independent samples considered in each group is indicated below the box plots.

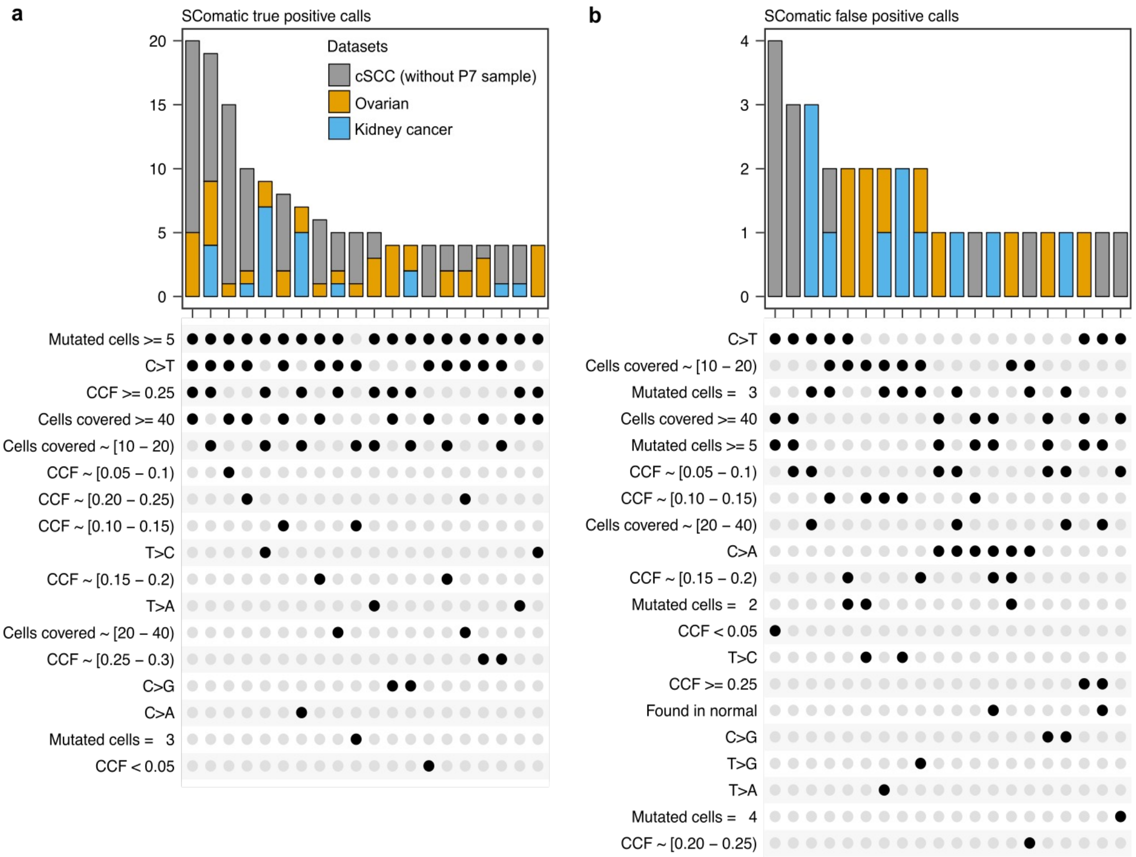

Supplementary Figure 7. Analysis of the properties of true (a) and false (b) positive calls computed using SComatic. Mutations were annotated for the cancer cell fraction (CCF) in the scRNA-seq data, nucleotide change, the total number of cells with sequencing coverage (Cells covered), the number of cells harbouring the mutation (Mutated cells), and the presence of supporting reads in WES/WGS data from the matched normal sample (Found in normal).

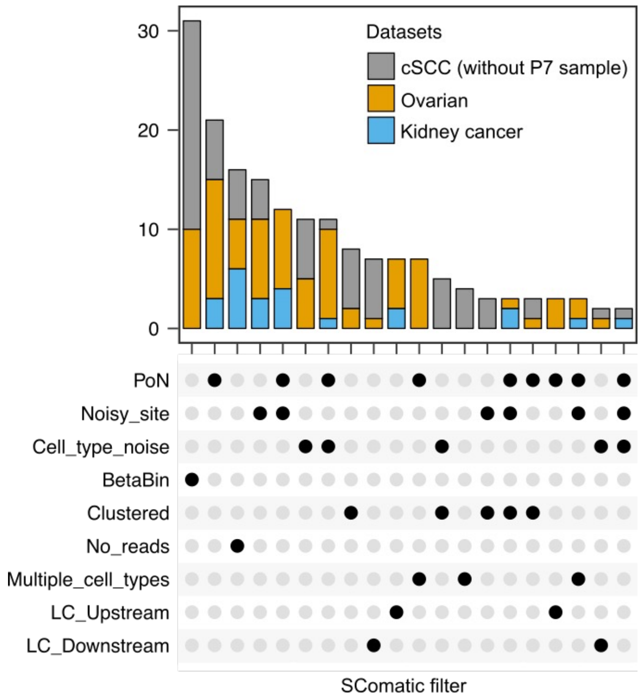

Supplementary Figure 8. Number of false negative calls in the benchmarking analysis stratified based on the filters applied by SComatic that were not satisfied. ***BetaBin***: the candidate mutation was not supported by a sufficient number of reads with the alternate allele to pass the Beta-binomial test; ***Cell_type_noise***: the number of reads supporting the alternate allele is only significant (Beta-binomial test) when applied to all cells across all cell types considered, but not when when applied to each cell type individually, or there are multiple alternate alleles, which suggests a noisy site; ***Clustered***: the candidate mutation was filtered because another candidate mutation maps within 5bp; ***LC_Upstream***: the candidate mutation was filtered because it mapped upstream of a low-complexity region; ***LC_Dowstream***: the candidate mutation was filtered because it mapped downstream of a low-complexity region; ***Multiple_cell_types***: the variant was found in different cell types of the same sample; ***No_reads***: no reads supporting the alternative allele were found; ***Noisy_site***: the candidate mutation filtered because there are a significant number of reads supporting the alternate allele in a single cell type when running the Beta-binomial test for each cell type independently, but the site is also significant when applying the Beta-binomial test to all single cells across all cell types in a sample together; ***PoN:*** variant filtered by the SComatic Panel of Normals (PoNs).

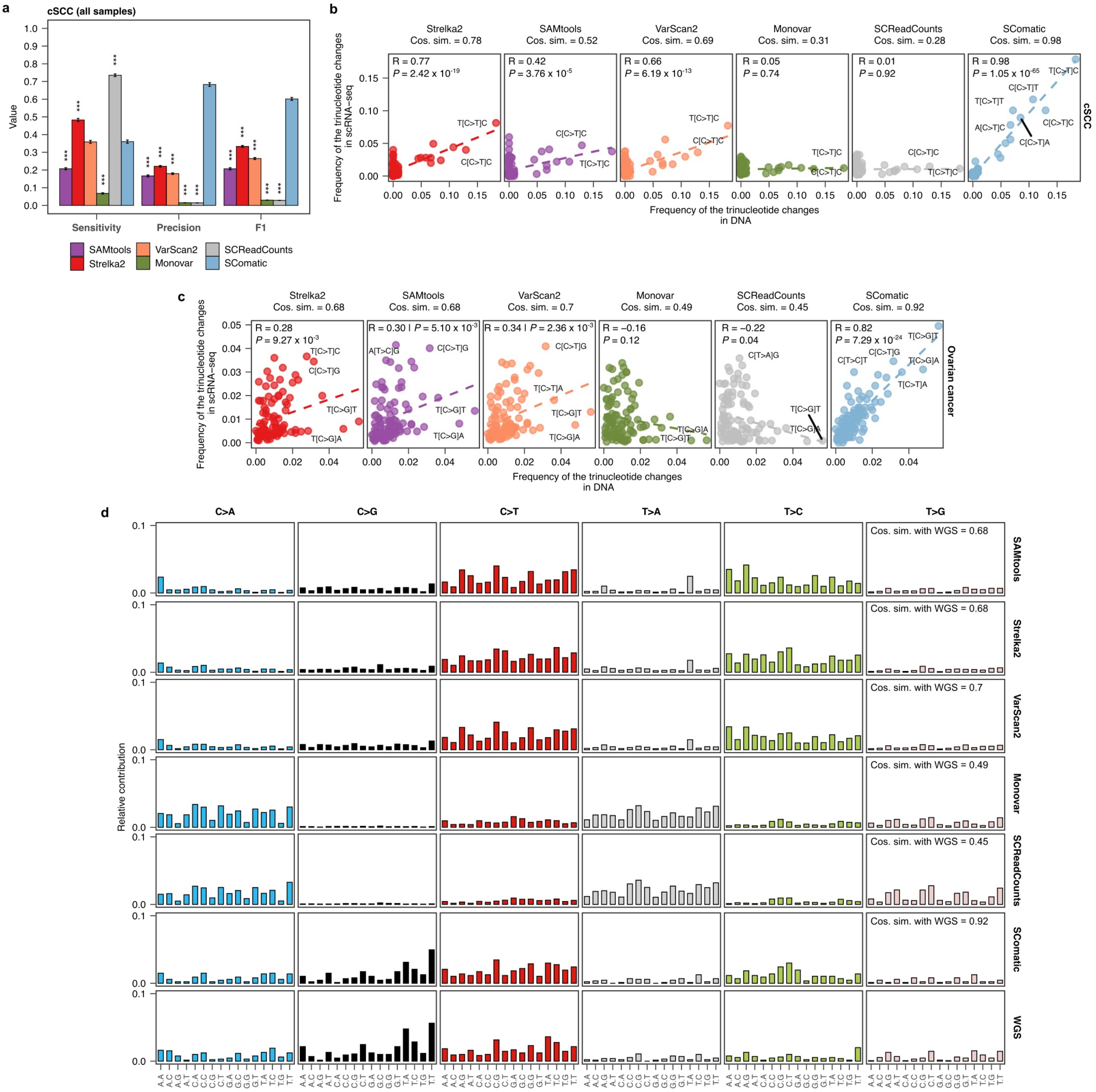

Supplementary Figure 9. Extended comparison of the performance of SComatic against other mutation detection methods. (**a**) Performance of Strelka2, SAMtools, VarScan2, Monovar, SCReadCounts and SComatic for the detection of somatic mutations in the scRNA-seq data from cSCC samples including sample P7. The error bars show the 95% bootstrap confidence interval for each statistic computed using 50 bootstrap resamples. Significance with respect to SComatic was assessed by the two-sided Student's t-test; ***$P < 10^{-15}$. (**b**) Correlation between the fraction of mutations detected in each trinucleotide context using the WES and scRNA-seq data from cSCC samples. (**c**) Correlation between the fraction of mutations detected in each trinucleotide context using the WGS and scRNA-seq data from ovarian cancer samples. Pearson correlations, FDR-adjusted P values, and cosine similarity values between the mutational spectra computed using the mutations detected in the scRNAs-seq and the WES/WGS data are shown in (**b**) and (**c**). (**d**) Comparison between the mutational spectra of the mutations detected in ovarian cancer samples using matched WGS and scRNA-seq data for each of the algorithms benchmarked. Cosine similarity values between the mutational spectra computed using the mutations detected in the scRNAs-seq and the WGS data are shown.

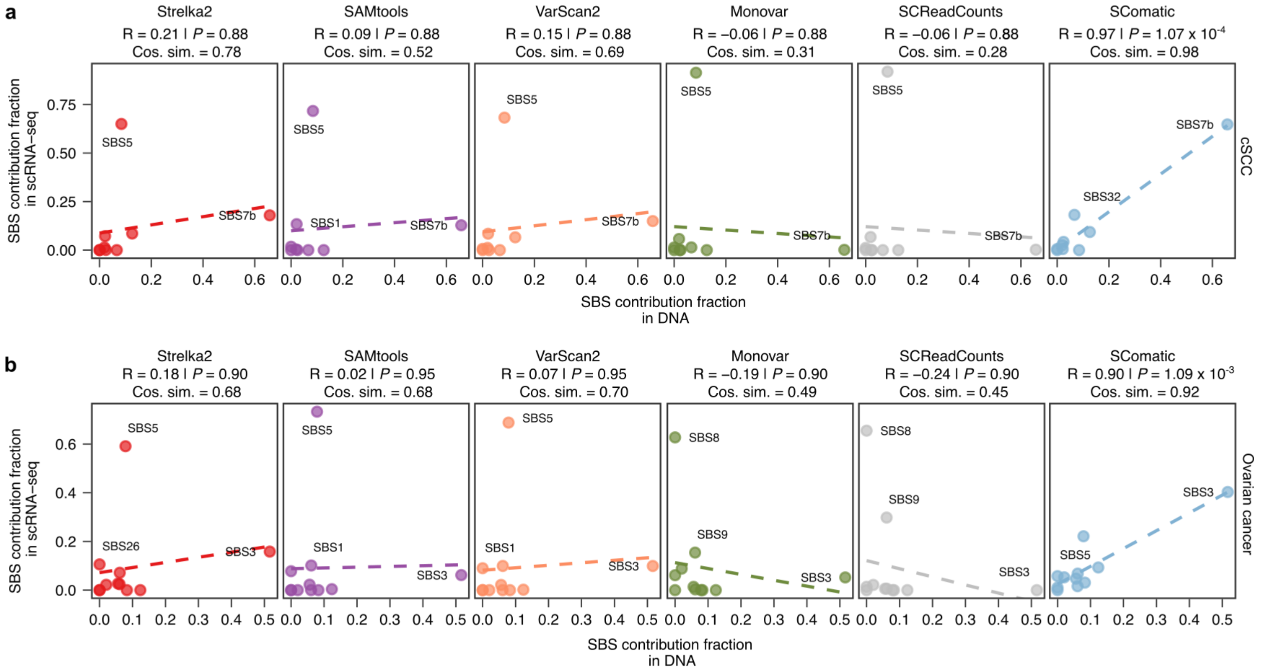

Supplementary Figure 10. Correlation between the estimated signature contributions computed using the mutations detected in WES/WGS (x axis) and scRNA-seq data (y-axis). FDR correction was applied to all *P* values reported. "Cos. sim.": cosine similarity values between the observed and the reconstructed mutational spectra using the estimated signature contributions.

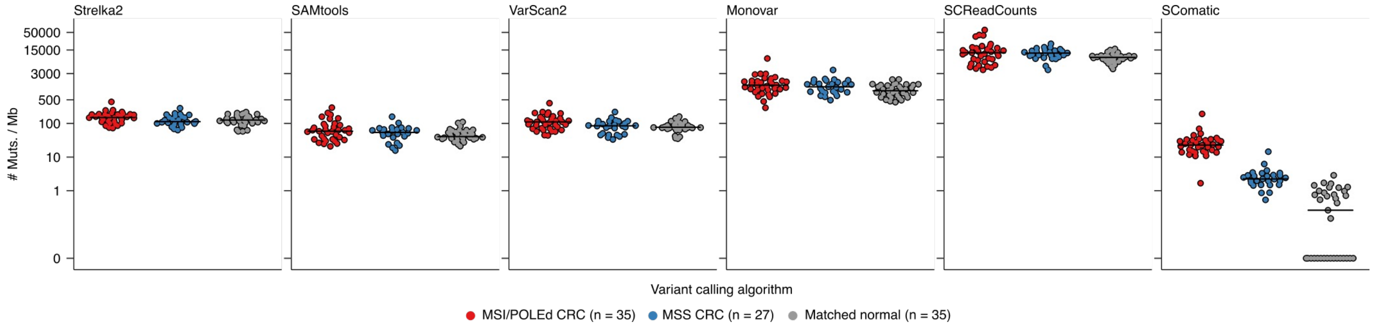

Supplementary Figure 11. Comparison of the mutational burden of epithelial cells computed using the mutations detected by Strelka2, SAMtools, VarScan2, Monovar, SCReadCounts and SComatic using the scRNA-seq data from colorectal cancers. Each dot represents a sample, and the black horizontal line shows the median for each group. The number of samples (n) per group is indicated in the legend.

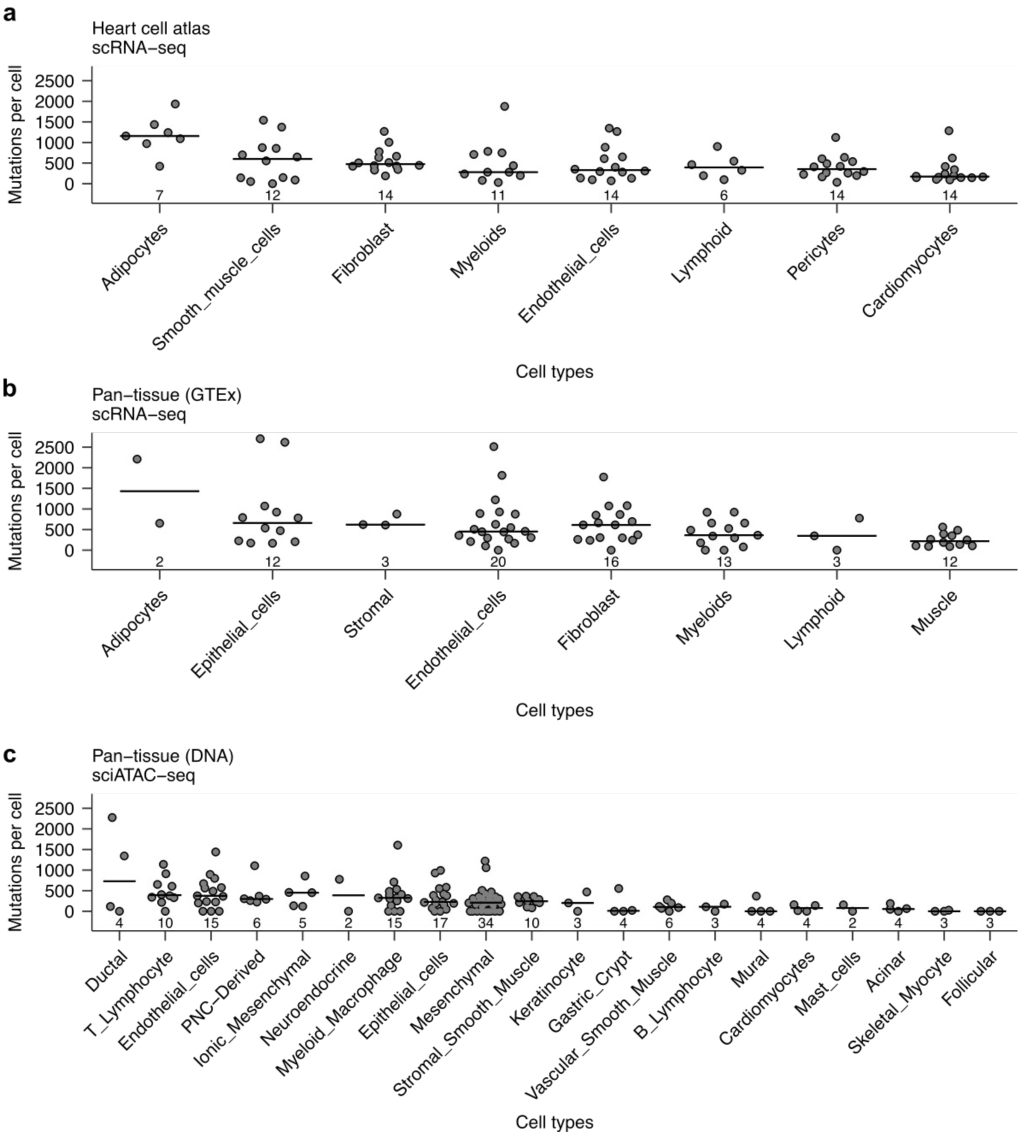

Supplementary Figure 12. Mutational burdens estimated for single cells. Average mutational burden for single cells across the cell types detected in the scRNA-seq data from **(a)** the heart cell atlas, **(b)** pan-tissue GTEx, and **(c)** pan-tissue sciATAC-seq data sets. Each dot represents the average number of mutations estimated for each cell per sample. Only samples with at least 100 cells per cell type and datasets with at least two samples are shown. The horizontal line shows the median value across samples. The number of individuals per cell type is indicated below the distributions.

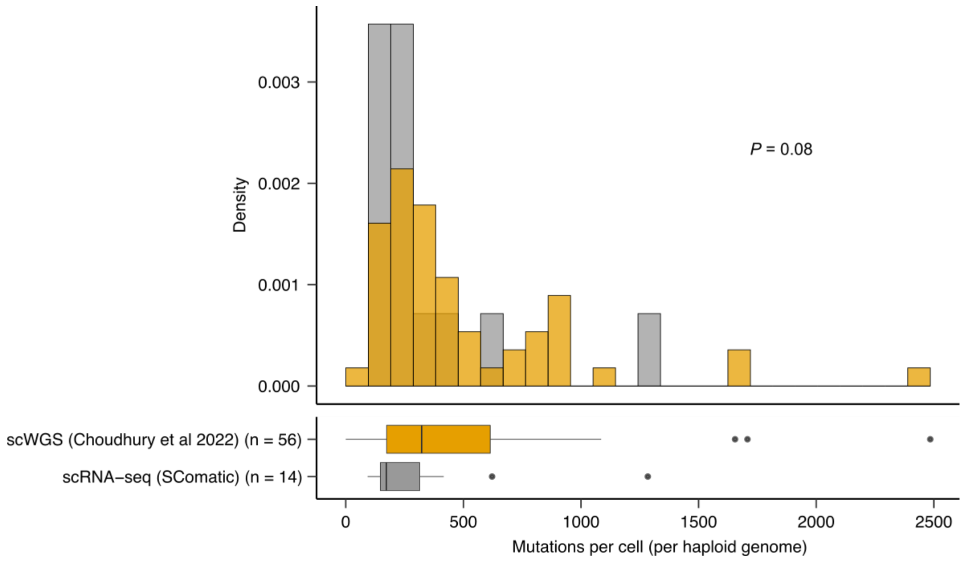

Supplementary Figure 13. Comparison of the mutation burdens estimated for cardiomyocytes using scRNA-seq and scWGS data. Mutation burdens per cardiomyocyte are shown and are normalised to mutations Mb and haploid genome. Mutation burdens estimated using scWGS data were divided by the ploidy of each single cell to compute haploid mutation loads. Data were taken from Choudhury et al., Nature Aging, 2022. The box plots show the median, first and third quartiles (boxes), and the whiskers encompass observations within a distance of 1.5x the interquartile range from the first and third quartiles. The sample size ("n") indicates the number of cardiomyocytes analysed in each group. Statistical significance was assessed using the two-sided Wilcoxon test.

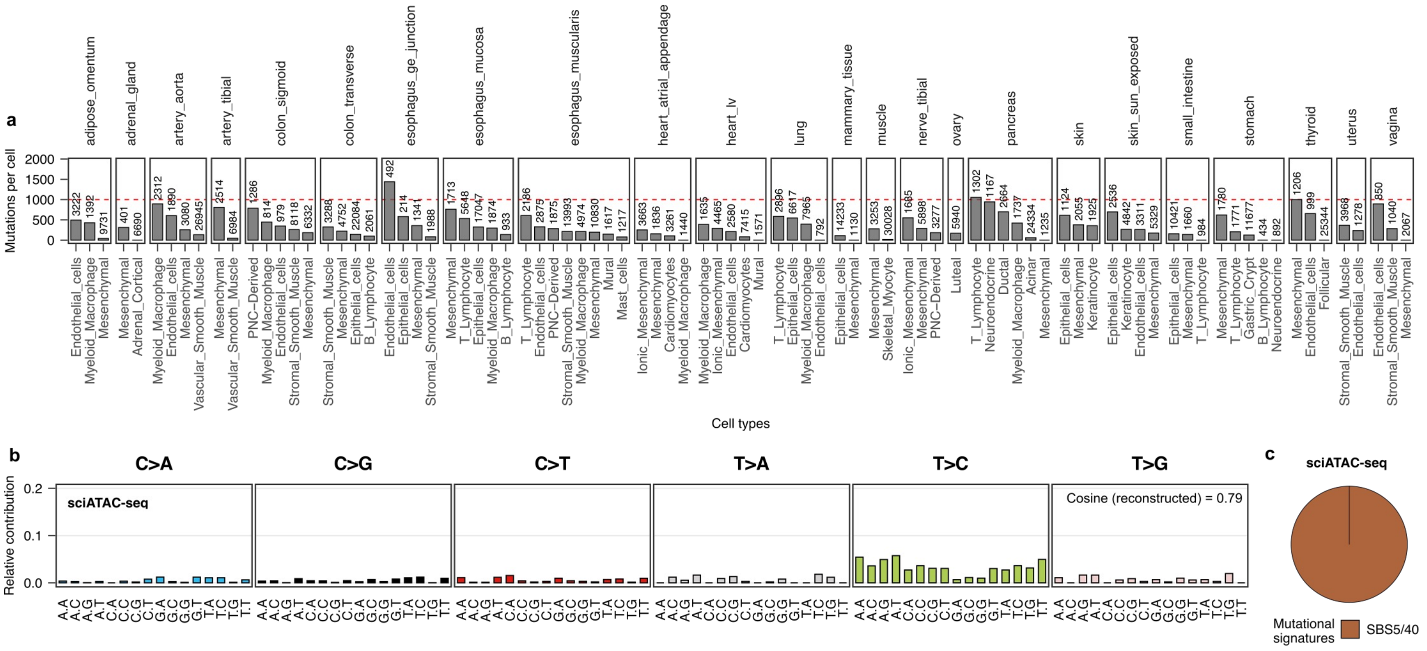

Supplementary Figure 14. Detection of somatic mutations in sciATAC-seq data using SComatic. (**a**) Average mutational burdens at the single cell level estimated using the somatic mutations detected by SComatic in sciATAC-seq data. The number on top of the bars indicates the number of cells per cell type. (**b**) Trinucleotide context of mutations detected across all cell types in the sciATAC-seq dataset. (**c**) Decomposition of the mutations detected in sciATAC-seq data across all cell types into COSMIC signatures (reconstructed cosine similarity = 0.79). The contributions of SBS5 and SBS40 are collapsed for visualization purposes.

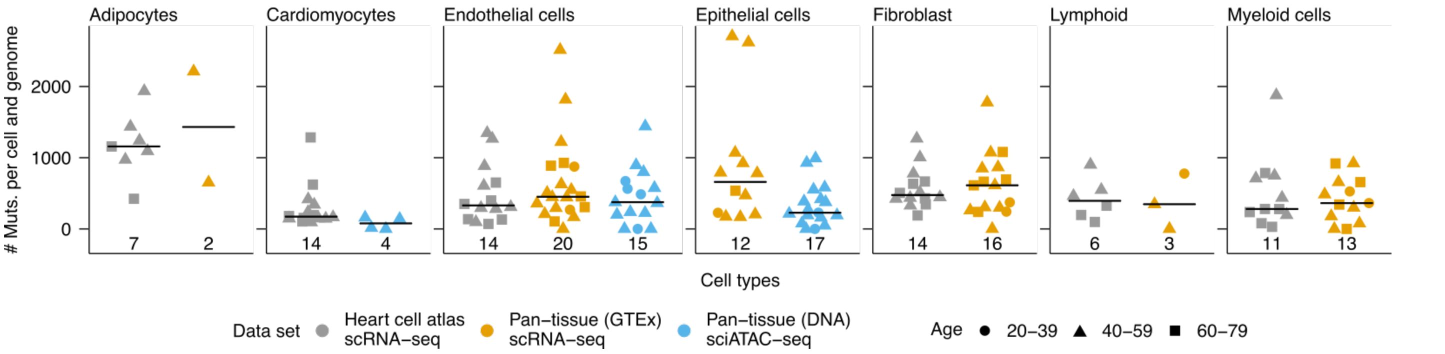

Supplementary Figure 15. Comparison of the mutational burdens per cell estimated for diverse cell types across datasets. Each dot represents the average number of mutations detected per cell and haploid genome for each donor. The horizontal line shows the median value across samples. Only datasets with at least two samples and cell types present in at least two datasets are shown. The number of independent samples per cell type is shown below the distributions.

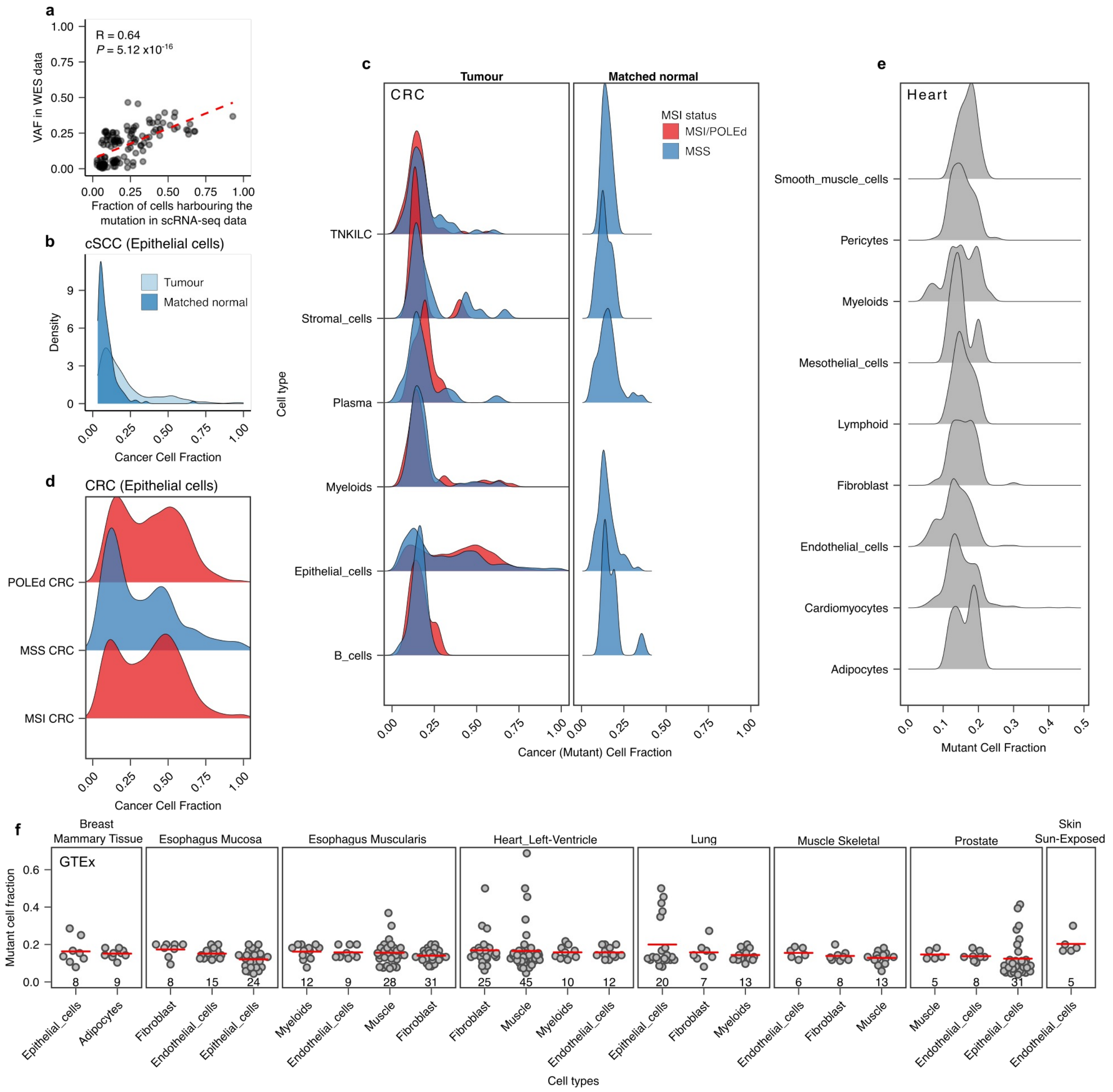

Supplementary Figure 16. Clonality of the mutations detected in scRNA-seq data. (**a**) Pearson correlation between the VAF of somatic mutations in WES and the fraction of cells harbouring the mutation in scRNA-seq data from the cSCC samples. The correlation and its significance was assessed using the Pearson's correlation test. Distribution of the cancer cell fraction values for mutations detected in scRNA-seq data from (**b**) cSCC tumours and matched normal skin samples, (**c**) colorectal tumours and matched normal samples, and (**d**) epithelial cells from colorectal tumour samples. Distribution of the cell fraction of mutations detected across cell types from (**e**) the heart cell atlas, and (**f**) the GTEx data set. Each dot in (**f**) represents an individual SNV and the red horizontal line shows the mean for each group. The number of independent samples per cell type is indicated below the distributions.

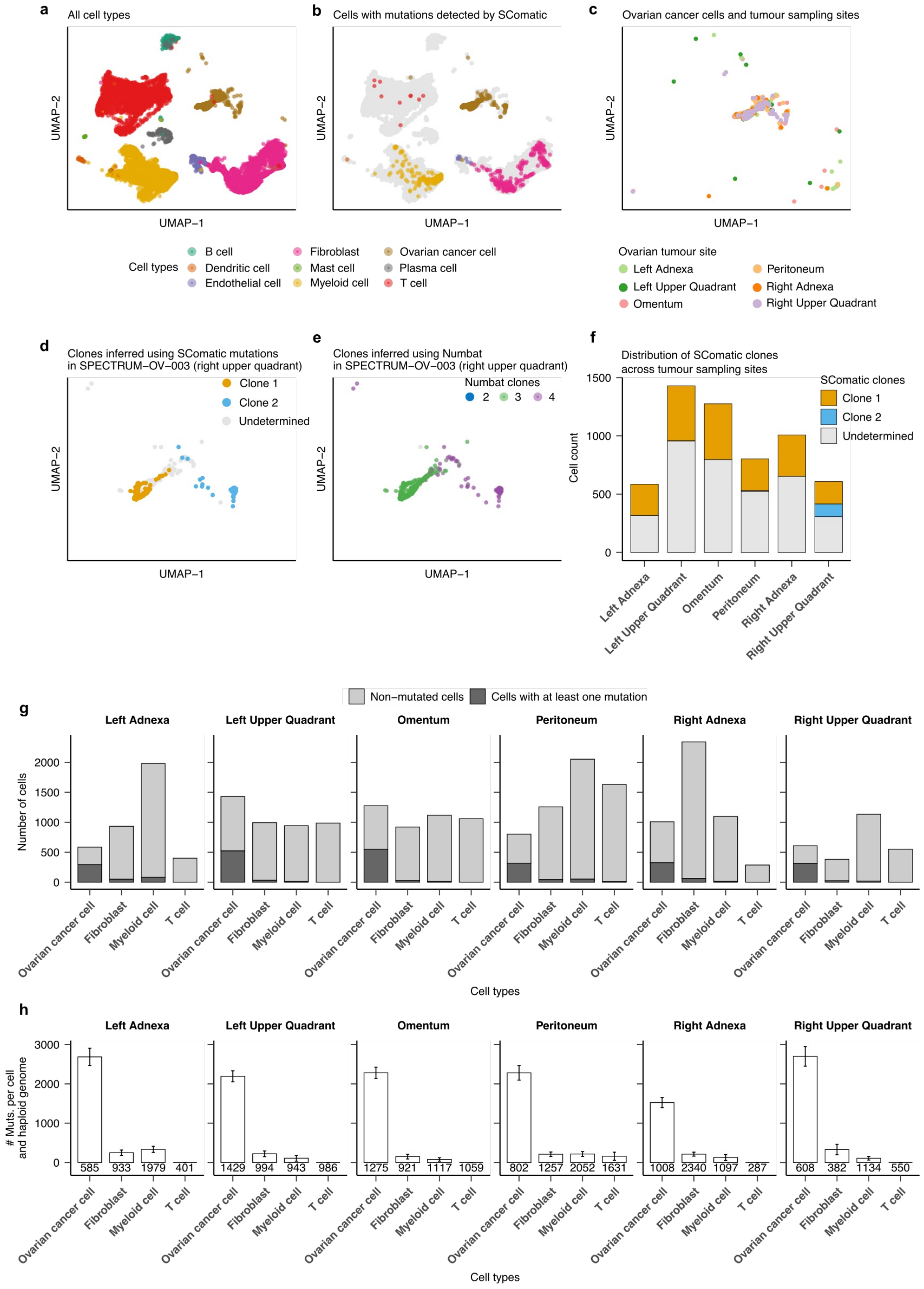

Supplementary Figure 17. Distribution of somatic mutations detected by SComatic in single cells from ovarian cancer samples. (**a**) Uniform Manifold Approximation and Projection (UMAP) coordinates of the cells detected using scRNA-seq data from six tumour regions from the ovarian cancer patient SPECTRUM-OV-003. Colours correspond to cell types and each dot represents a single cell. (**b**) Projection of somatic mutations. Coloured dots mark single cells in which a somatic mutation was detected. The colour indicates the cell type in which the mutation was detected. Grey dots correspond to single cells in which no mutation was detected. (**c**) UMAP coordinates of the cells classified as malignant. Colours indicate the tumour regions from which cells were collected. (**d**) Same as (**c**) with cells coloured based on clone assignments defined by mutations detected by SComatic. (**e**) Same as (**c**) with cells coloured according to clone assignments generated by Numbat based on copy number profiles inferred from the same scRNA-seq data used to detect mutations using SComatic. (**f**) Fraction of cells from each tumour region that were assigned to the clones defined by the mutations detected by SComatic. (**g**) Number of cells of each cell type in which at least one somatic mutation was detected by SComatic across different ovarian tumour regions profiled using scRNA-seq. Only cell types with at least 100 cells per sampling site are shown. (**h**) Estimated number of mutations per haploid genome per cell. The bar plots show the average number of mutations across all cells of the same type and 95% confidence intervals. Only cell types with at least 100 cells per sampling site are shown. The number shown below each bar represents the number of cells per cell type.

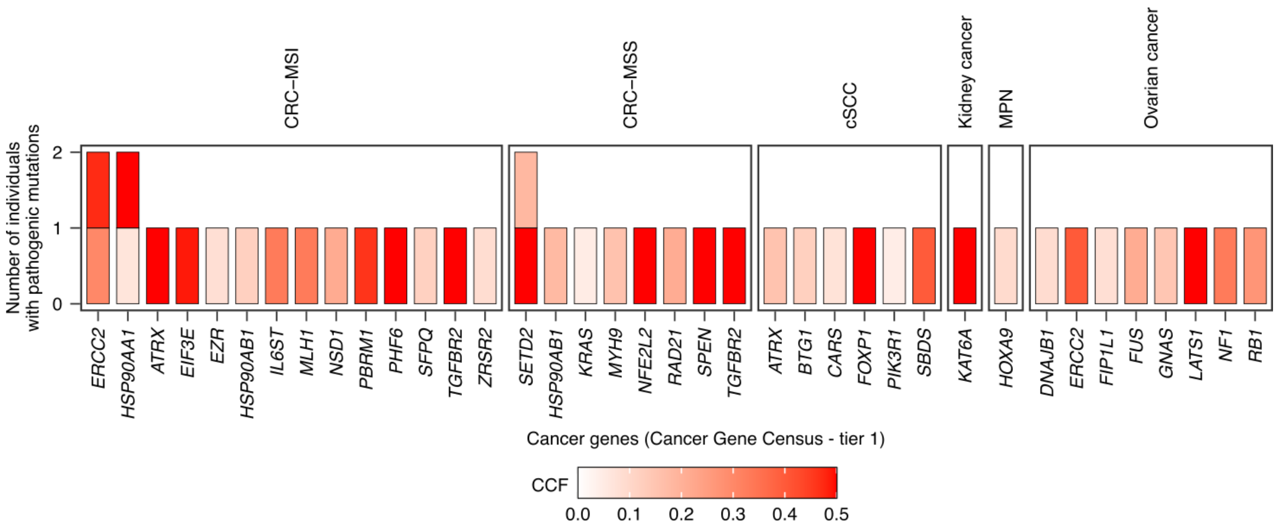

Supplementary Figure 18. Somatic mutations detected by SComatic in cancer-associated genes and predicted to be deleterious. The colour bar indicates the CCF of the mutations in the scRNA-seq data.

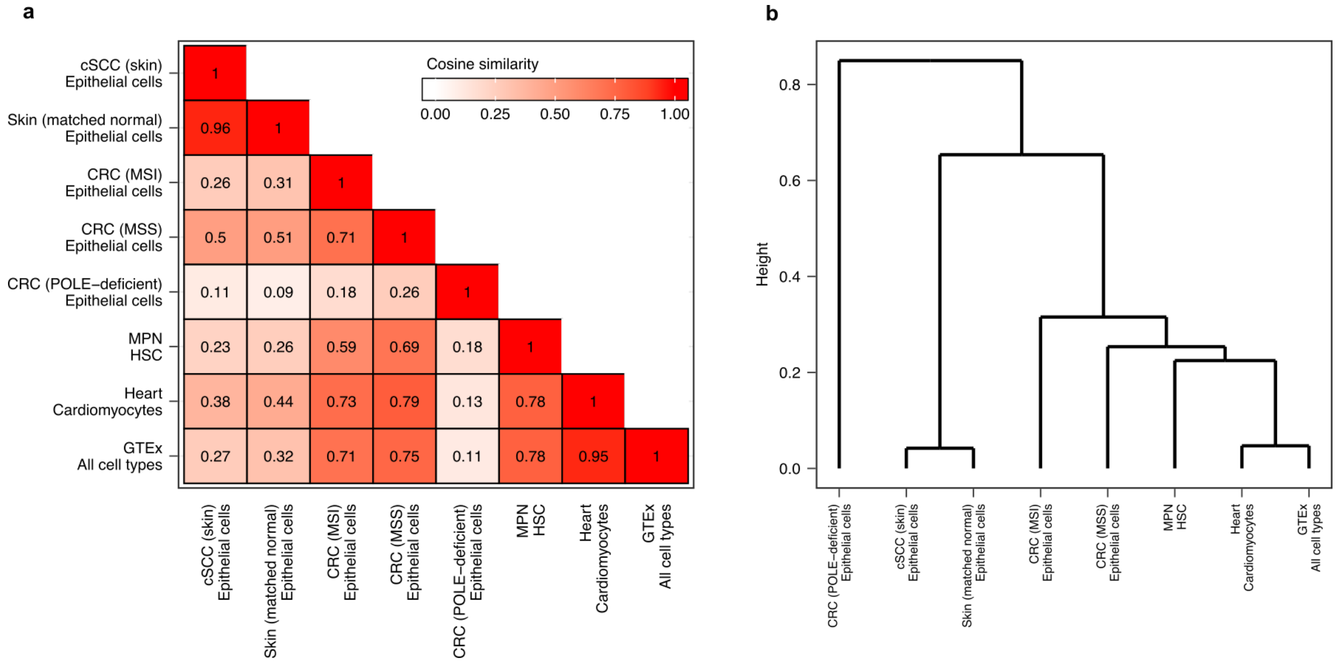

Supplementary Figure 19. Comparison of the mutational patterns detected across the data sets analysed in this study. (**a**) Pairwise cosine similarities between the mutational spectra computed using the mutations detected across cell types from each data set. (**b**) Hierarchical clustering based on the cosine similarity comparison (shown in **a**) of the mutational spectra detected in each data set using SComatic.

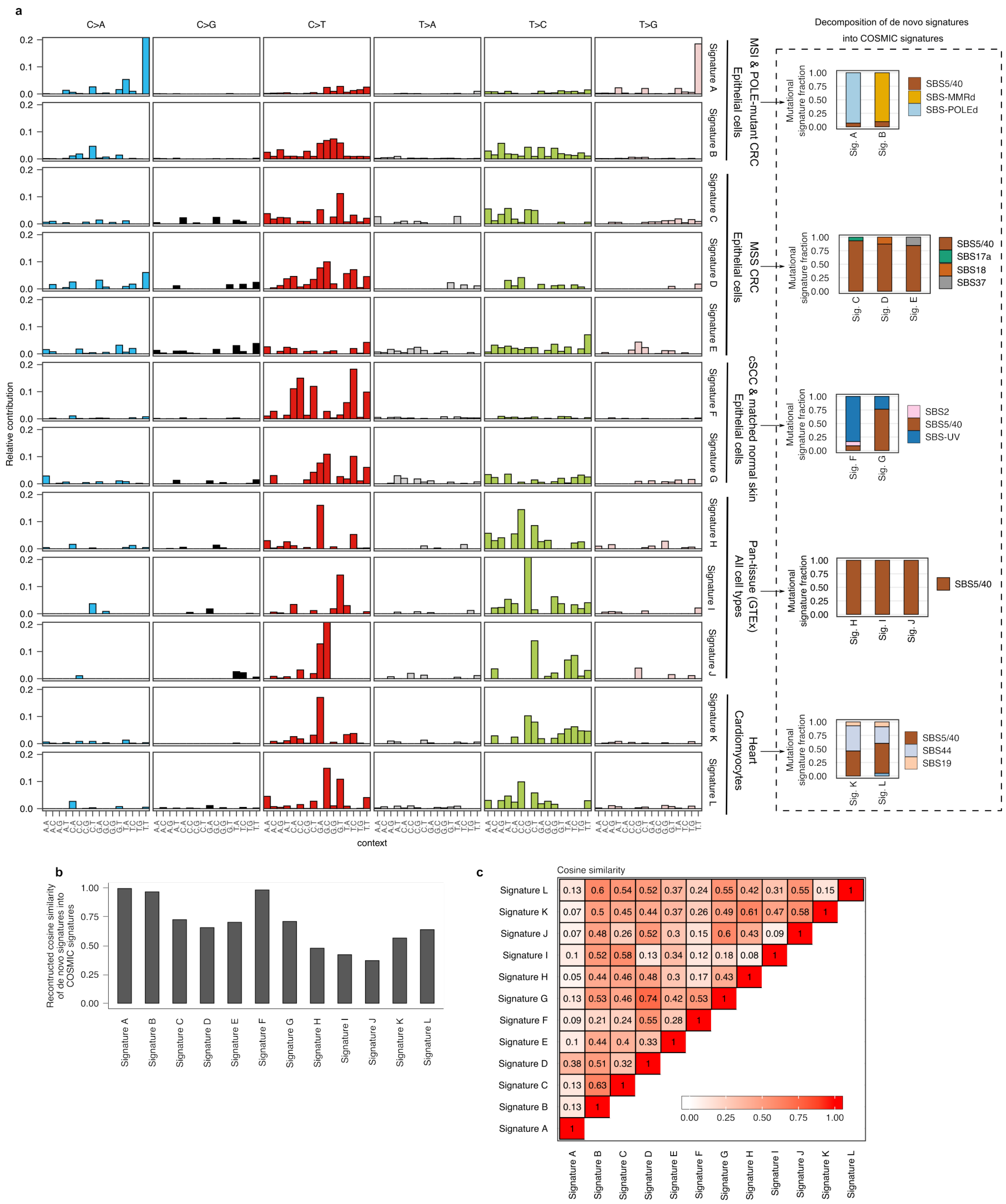

Supplementary Figure 20. *De novo* mutational signature analysis of the somatic mutations discovered by SComatic. (**a**) Trinucleotide context of the *de novo* signatures discovered in the data sets analysed. *De novo* mutational signatures were extracted independently from each dataset. The decomposition of the *de novo* signatures into COSMIC signatures was also run for each dataset independently. (**b)** Cosine similarities between the *de novo* signatures and the reconstructed mutational spectra using the estimated signature contributions. (**c**) Pairwise cosine similarities between each pair of mutational signatures extracted *de novo*. Mutational signatures associated with MMRd (SBS6, SBS14, SBS15, SBS21, SBS26 and SBS44), POLE deficiency (SBS10a, SBS10b and SBS28), ultraviolet radiation (SBS7a,b,c and d) and clock-like mutational processes (SBS5 and SBS40) are collapsed for visualization purposes.

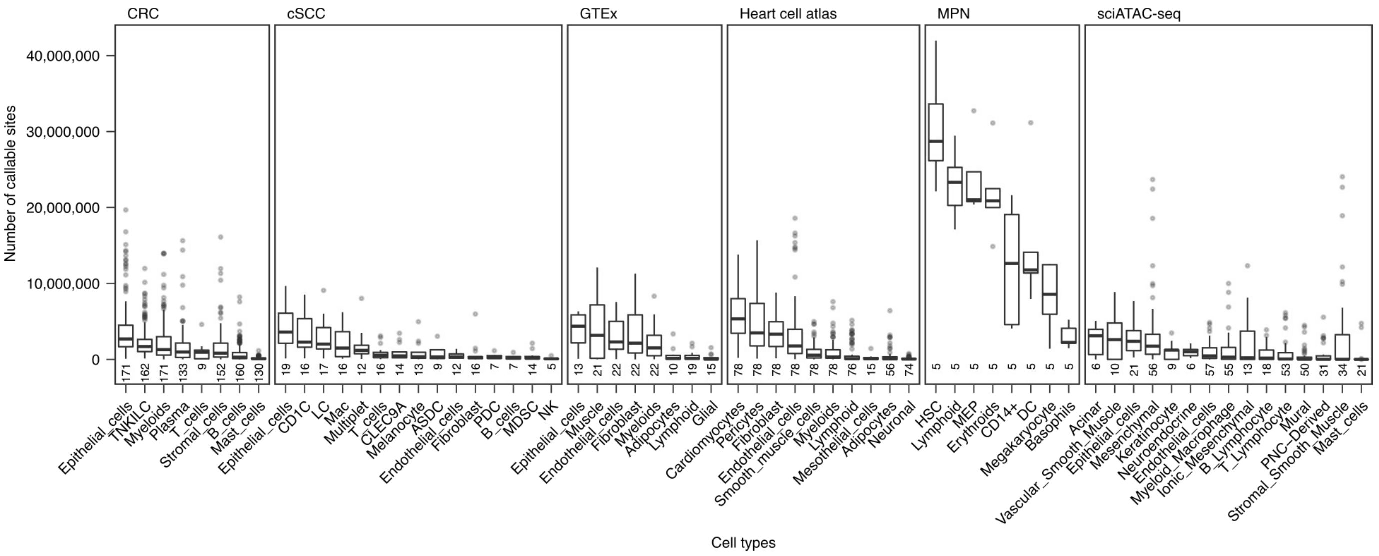

Supplementary Figure 21. Number of callable sites per cell type and data set. The box plots show the median, first and third quartiles (boxes), and the whiskers encompass observations within a distance of 1.5x the interquartile range from the first and third quartiles. The number of samples per group is shown below the box plots.
