# Supplementary material for: De novo detection of somatic mutations in high-throughput single-cell profiling data sets
Source: Nat Biotechnol. 2023 Jul 6;42(5):758–67. doi: 10.1038/s41587-023-01863-z (PMC11098751; doi:10.1038/s41587-023-01863-z)
Supplement: Supplementary file 2 — Reporting Summary [file 41587_2023_1863_MOESM2_ESM.pdf]

Corresponding author(s): Isidro Cortes-Ciriano, PhD

Last updated by author(s): May 26, 2023

Reporting Summary

Nature Portfolio wishes to improve the reproducibility of the work that we publish. This form provides structure for consistency and transparency in reporting. For further information on Nature Portfolio policies, see our Editorial Policies and the Editorial Policy Checklist.

Statistics

For all statistical analyses, confirm that the following items are present in the figure legend, table legend, main text, or Methods section.

n/a	Confirmed	
☐	☒	The exact sample size (*n*) for each experimental group/condition, given as a discrete number and unit of measurement
☒	☐	A statement on whether measurements were taken from distinct samples or whether the same sample was measured repeatedly
☐	☒	The statistical test(s) used AND whether they are one- or two-sided<br>*Only common tests should be described solely by name; describe more complex techniques in the Methods section.*
☒	☐	A description of all covariates tested
☐	☒	A description of any assumptions or corrections, such as tests of normality and adjustment for multiple comparisons
☐	☒	A full description of the statistical parameters including central tendency (e.g. means) or other basic estimates (e.g. regression coefficient) AND variation (e.g. standard deviation) or associated estimates of uncertainty (e.g. confidence intervals)
☐	☒	For null hypothesis testing, the test statistic (e.g. *F*, *t*, *r*) with confidence intervals, effect sizes, degrees of freedom and *P* value noted<br>*Give P values as exact values whenever suitable.*
☒	☐	For Bayesian analysis, information on the choice of priors and Markov chain Monte Carlo settings
☒	☐	For hierarchical and complex designs, identification of the appropriate level for tests and full reporting of outcomes
☐	☒	Estimates of effect sizes (e.g. Cohen's *d*, Pearson's *r*), indicating how they were calculated

Our web collection on statistics for biologists contains articles on many of the points above.

Software and code

Policy information about availability of computer code

Data collection

Data collection was performed using the UNIX command line. No other software was used for data collection.

Data analysis

All the single-cell RNA-seq data sets analysed in the study were aligned to the reference genome using Cell Ranger version 6.0.1. The ATAC-seq data sets were aligned to the reference genome using BWA-MEM v0.7.17-r1188.

The following software packages have been used for data analysis in this study:

SComatic dependencies:
- Python version >=3.7.0
- Python packages:
About-time (version 3.1.1)
numpy (version 1.21.6)
numpy-groupies (version 0.9.14)
pandas (version 1.3.5)
pybedtools (version 0.8.1)
pysam (version 0.16.0.1)
rpy2 (version 2.9.4)
scipy (version 1.7.3)
- R version >=3.6.0
- R package VGAM (version 1.1-5)
- SAMtools (version >=1.11)

- BEDtools (version >=v2.30.0)
- Datamash (version >=v1.1.0)

Other software used in the manuscript:
- Genome Analysis Toolkit (GATK) (version v4.1.8.0)
- BWA-MEM (version v0.7.17-r1188)
- Cell Ranger (version 6.0.1)
- R package MutationalPatterns (version 1.12.0)
- SigMA (version v1)
- Strelka2 (version 2.9.10)
- MuSE (version 1.0rc)
- SAGE (version v2.8)
- PURPLE (version v2.54)
- VarScan2 (version v2.3.9)
- Monovar (version v0.0.1)
- SCReadCounts (version 1.3.2)
- SAMtools (version v1.11)
- BEDtools (version v2.30.0)
- cellsnp-lite (version 1.2.2)
- Eagle2 (version v2.4.1)
- Numbat (version 1.2.1)
- Annovar (version 2018Apr16)

The code to reproduce the analyses presented in this study is available at https://github.com/cortes-ciriano-lab/SComatic

For manuscripts utilizing custom algorithms or software that are central to the research but not yet described in published literature, software must be made available to editors and reviewers. We strongly encourage code deposition in a community repository (e.g. GitHub). See the Nature Portfolio guidelines for submitting code & software for further information.

Data

Policy information about availability of data

All manuscripts must include a data availability statement. This statement should provide the following information, where applicable:

- Accession codes, unique identifiers, or web links for publicly available datasets
- A description of any restrictions on data availability
- For clinical datasets or third party data, please ensure that the statement adheres to our policy

The raw WES and scRNA-seq data from the skin squamous cell carcinoma and matched normal samples are available at the Gene Expression Omnibus (GEO) database under the accession number GSE144240. The raw scRNA-seq data from MPN and colorectal cancer patients are available through controlled access application via dbGaP under dbGaP Study Accession numbers phs002308.v1.p1 and phs002407.v1.p1, respectively. The cell type annotations for the colorectal cancer data set36 are available at GEO database under the accession number GSE178341. Raw sequencing data and cell type annotations for 6 additional colorectal cancer patients37 included in this study are available at GEO database under the accession number GSE144735. The cell type annotations for the MPN data set were obtained from our previous study8. Raw WES sequencing and scRNA-seq data from the kidney cancer samples are available at the European Genome-Phenome Archive (EGA) under accession numbers: EGAD00001008029 and EGAD00001008030, respectively. Cell type annotations can be accessed at https://data.mendeley.com/datasets/g67bkbnhhg/1. Instructions to access the whole-genome sequencing data, scRNA-seq data and somatic copy number calls from the ovarian cancer data set are available at https://www.synapse.org/#!Synapse:syn25569736/wiki/612269. The raw scRNA-seq data and cell type annotations for the human heart cell atlas44 were downloaded from the Human Cell Atlas Data Coordination Platform with accession number ERP123138 (https://www.ebi.ac.uk/ena/browser/view/ERP123138). Cell type annotations were downloaded from the Human Cell Atlas Data Portal (https://data.humancellatlas.org/explore/projects/ad98d3cd-26fb-4ee3-99c9-8a2ab085e737). The raw single-cell ATAC-seq data and cell type annotations used in this study are available at GEO database under the accession number GSE184462. The raw sequencing data from GTEx samples are available at the Analysis Visualization and Informatics Lab-space (AnVIL; https://anvil.terra.bio/#workspaces/anvil-datastorage/AnVIL_GTEx_V9_hg38) and can be downloaded through controlled data access application via dbGaP under Study Accession number: phs000424. The gnomAD database (version 2.0.1) and the ExAC data set (version 0.3) were downloaded from https://gnomad.broadinstitute.org. The COSMIC mutational signatures (version 3) were downloaded from https://cancer.sanger.ac.uk/signatures/. The Cancer Gene Census (GCG) catalogue (version July-2022) was downloaded from https://cancer.sanger.ac.uk/census. The GATK Panel of Normals generated using WGS data from the 1000G project was downloaded from https://gatk.broadinstitute.org/hc/en-us/articles/360035890631-Panel-of-Normals-PON-. The DARNED database was downloaded from https://darned.ucc.ie. The REDIportal Database was downloaded from http://srv00.recas.ba.infn.it/atlas/download.html. Exome sequencing data from The Cancer Genome Atlas (TCGA) are available through controlled data access via dbGaP with accession number phs000178.v11.p8 and were downloaded from the Genomic Data Commons (GDC) Data Portal (https://portal.gdc.cancer.gov). The reference genome used in this study (GRCh38) was downloaded from https://hgdownload.soe.ucsc.edu/downloads.html.

Human research participants

Policy information about studies involving human research participants and Sex and Gender in Research.

Reporting on sex and gender	NA
Population characteristics	NA
Recruitment	NA

Ethics oversight | NA

Note that full information on the approval of the study protocol must also be provided in the manuscript.

Field-specific reporting

Please select the one below that is the best fit for your research. If you are not sure, read the appropriate sections before making your selection.

- [x] Life sciences
- [ ] Behavioural & social sciences
- [ ] Ecological, evolutionary & environmental sciences

For a reference copy of the document with all sections, see nature.com/documents/nr-reporting-summary-flat.pdf

Life sciences study design

All studies must disclose on these points even when the disclosure is negative.

Sample size	We collected and uniformly processed published scRNAs-seq data from 622 samples and scATAC-seq data from 66 samples, totalling 2,655,775 non-neoplastic and cancer cells.
Data exclusions	We did not exclude any data points from the published data sets analysed.
Replication	The main findings in this study are related to the discovery of somatic mutations in single-cell data sets using a new algorithm, SComatic. To assess the performance of the algorithm to detect mutations, we analysed multiple data sets and single-cell technologies.
Randomization	No specific allocation of samples was performed.
Blinding	This study did not involve recruitment of patients.

Reporting for specific materials, systems and methods

We require information from authors about some types of materials, experimental systems and methods used in many studies. Here, indicate whether each material, system or method listed is relevant to your study. If you are not sure if a list item applies to your research, read the appropriate section before selecting a response.

Materials & experimental systems

n/a	Involved in the study	
[x]	[ ]	Antibodies
[x]	[ ]	Eukaryotic cell lines
[x]	[ ]	Palaeontology and archaeology
[x]	[ ]	Animals and other organisms
[x]	[ ]	Clinical data
[x]	[ ]	Dual use research of concern

Methods

n/a	Involved in the study	
[x]	[ ]	ChIP-seq
[x]	[ ]	Flow cytometry
[x]	[ ]	MRI-based neuroimaging
